# Exploring Structure and Function of Redox Intermediates in [NiFe]‐Hydrogenases by an Advanced Experimental Approach for Solvated, Lyophilized and Crystallized Metalloenzymes

**DOI:** 10.1002/anie.202100451

**Published:** 2021-05-05

**Authors:** Christian Lorent, Vladimir Pelmenschikov, Stefan Frielingsdorf, Janna Schoknecht, Giorgio Caserta, Yoshitaka Yoda, Hongxin Wang, Kenji Tamasaku, Oliver Lenz, Stephen P. Cramer, Marius Horch, Lars Lauterbach, Ingo Zebger

**Affiliations:** ^1^ Department of Chemistry Technische Universität Berlin Strasse des 17. Juni 135 10623 Berlin Germany; ^2^ Japan Synchrotron Radiation Research Institute SPring-8 1-1-1 Kouto, Mikazuki-cho Sayo-gun Hyogo 679-5198 Japan; ^3^ SETI Institute 189 Bernardo Avenue Mountain View California 94043 USA; ^4^ RIKEN SPring-8 center 1-1-1 Kouto, Sayo-cho Sayo-gun Hyogo 679-5148 Japan; ^5^ Department of Physics Freie Universität Berlin Arnimallee 14 14195 Berlin Germany

**Keywords:** [NiFe]-hydrogenase, biocatalysis, in situ spectroscopy, metalloenzymes, vibrational spectroscopy

## Abstract

To study metalloenzymes in detail, we developed a new experimental setup allowing the controlled preparation of catalytic intermediates for characterization by various spectroscopic techniques. The in situ monitoring of redox transitions by infrared spectroscopy in enzyme lyophilizate, crystals, and solution during gas exchange in a wide temperature range can be accomplished as well. Two O_2_‐tolerant [NiFe]‐hydrogenases were investigated as model systems. First, we utilized our platform to prepare highly concentrated hydrogenase lyophilizate in a paramagnetic state harboring a bridging hydride. This procedure proved beneficial for ^57^Fe nuclear resonance vibrational spectroscopy and revealed, in combination with density functional theory calculations, the vibrational fingerprint of this catalytic intermediate. The same in situ IR setup, combined with resonance Raman spectroscopy, provided detailed insights into the redox chemistry of enzyme crystals, underlining the general necessity to complement X‐ray crystallographic data with spectroscopic analyses.

## Graphical abstract

A multifunctional setup for in situ spectroscopy on gas‐processing metalloenzymes enables the controlled preparation of redox states in various sample forms. This setup allowed the first NRVS characterization of the Ni_a_−C state of [NiFe]‐hydrogenases, provided new insights into the reductive activation of these enzymes, and revealed a so‐far unknown redox state of the oxygen‐tolerant membrane‐bound hydrogenase from *R. eutropha*.

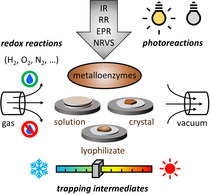

## Introduction

Transition metals are often involved in chemical and enzymatic catalysis. In nature, metal‐containing enzymes catalyze a variety of reactions, especially the conversion of small gaseous molecules like CO_2_, N_2_, or H_2_. This type of chemistry is relevant for establishing alternative strategies for energy conversion and the production of carbon‐neutral fuels. Many of these metalloenzymes, including carbon monoxide dehydrogenase, nitrogenase and hydrogenase, are attractive targets for biotechnological application and can serve as blueprints for bioinspired chemistry.[^1^, ^2^, ^3^, ^4^, ^5^, ^6^] However, their rational utilization requires a thorough mechanistic understanding, typically requiring multiple spectroscopic techniques.

Infrared (IR) spectroscopy in various implementations has been successfully used to monitor catalytic processes and intermediates at and beyond biological metal sites. Available approaches comprise surface‐sensitive techniques like surface‐enhanced infrared absorption (SEIRA) and/or attenuated total reflection (ATR) spectroscopy, optionally combined with electrochemistry, illumination, gas‐atmosphere and/or temperature control.[^7^, ^8^, ^9^, ^10^, ^11^, ^12^] Recently, time‐resolved IR studies in the nanosecond range as well as ultrafast pump‐probe and two‐dimensional IR techniques have also been introduced into metalloenzyme research.[^13^, ^14^] Electron paramagnetic resonance (EPR) spectroscopy provides additional structural and electronic insight into paramagnetic states,[^15^, ^16^, ^17^, ^18^] while resonance Raman (RR) spectroscopy has been successfully applied to monitor the characteristic metal−ligand modes of specific redox states and cofactors.[^19^, ^20^, ^21^, ^22^, ^23^, ^24^] Additionally, ^57^Fe nuclear resonance vibrational spectroscopy (NRVS), a synchrotron‐based technique that can selectively probe iron‐specific normal modes, has been lately established for characterizing iron‐containing enzymes.[^25^, ^26^, ^27^, ^28^, ^29^, ^30^, ^31^, ^32^]

Here, we have designed a new experimental setup for spectroscopic analyses of gas‐converting metalloenzymes in various sample forms. Gas composition and temperature can be adjusted, thereby enabling the preparation of specific redox states for subsequent characterization by complementary spectroscopic tools, such as RR, EPR, or NRVS. Additionally, redox transitions between catalytically relevant intermediates and resting states can be monitored by in situ IR spectroscopy.

**Figure 1:**
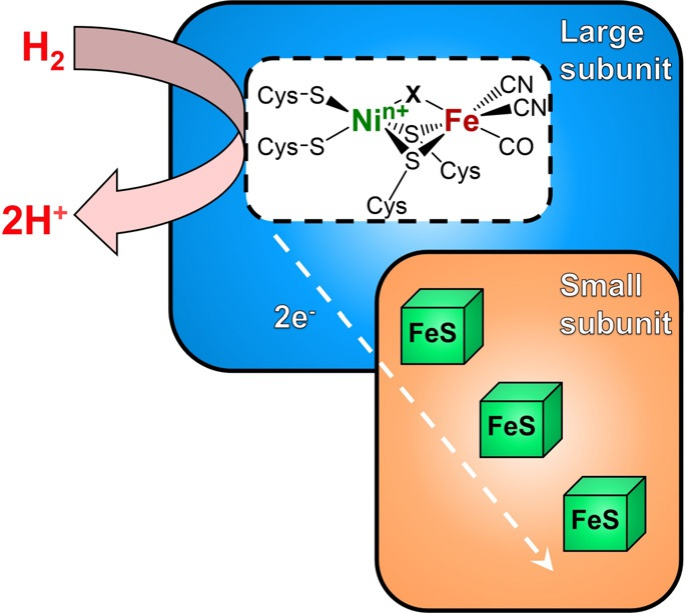
Schematic representation of the consensus structure and cofactor composition of the model [NiFe]‐hydrogenases investigated in this study. The redox state of the active site is primarily determined by the oxidation state of the nickel ion and the nature of the bridging ligand X.

To illustrate the versatility of our approach and its benefits for exploring metalloenzyme catalysis, we investigated two O_2_‐tolerant model enzymes, the regulatory and the membrane‐bound [NiFe]‐hydrogenase from *Ralstonia eutropha* (*Re*RH and *Re*MBH, respectively). Briefly, [NiFe]‐hydrogenases catalyze the reversible cleavage of molecular hydrogen into protons and electrons (Figure 1). All of these enzymes feature a heterodimeric core structure, consisting of a large subunit harboring the [NiFe] active site and a small subunit comprising iron–sulfur clusters that form an electron transfer relay. The proposed catalytic cycle and redox intermediates of [NiFe]‐hydrogenases discussed in this study are displayed in Figure S1.

## Results and Discussion

**Figure 2:**
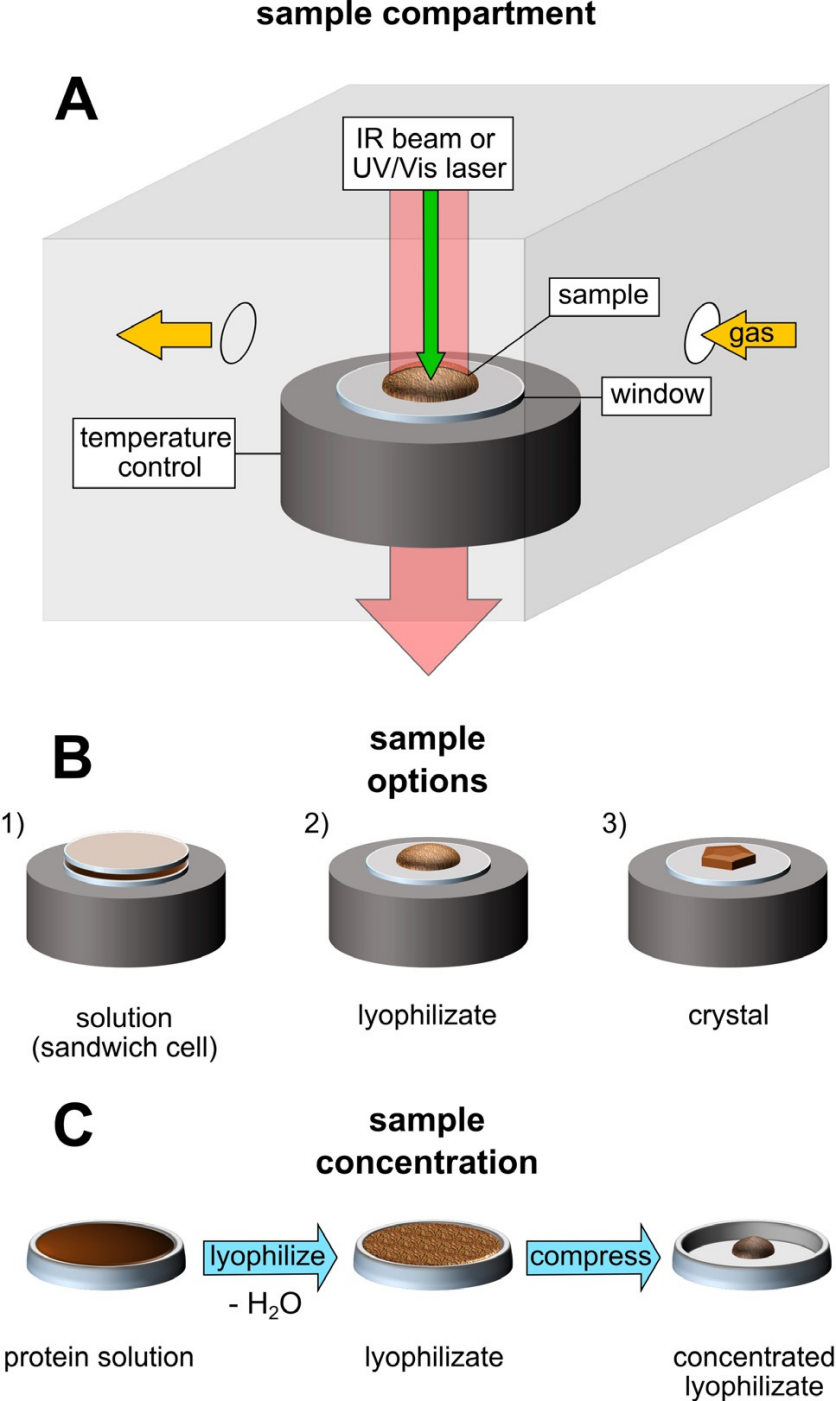
Setup for in situ IR analysis of proteins. A) The sample compartment consists of a gas‐tight, temperature‐controlled chamber with IR‐transparent windows. A gas supply allows the exchange of various dried or humidified gases (H_2_, N_2_, O_2_) and mixtures thereof. B) Protein samples can be studied in multiple forms: in solution in a sandwich cell made of two IR windows (Figures 5, S3B and S11B), as lyophilized powder (Figures 3 C and S3A) or in the crystalline phase (Figures 5 and S11A) on top of a single window. C) The concentration of a protein sample can be significantly increased, up to an equivalent of 4–5 mm, via lyophilization and subsequent compression.

**Figure 3:**
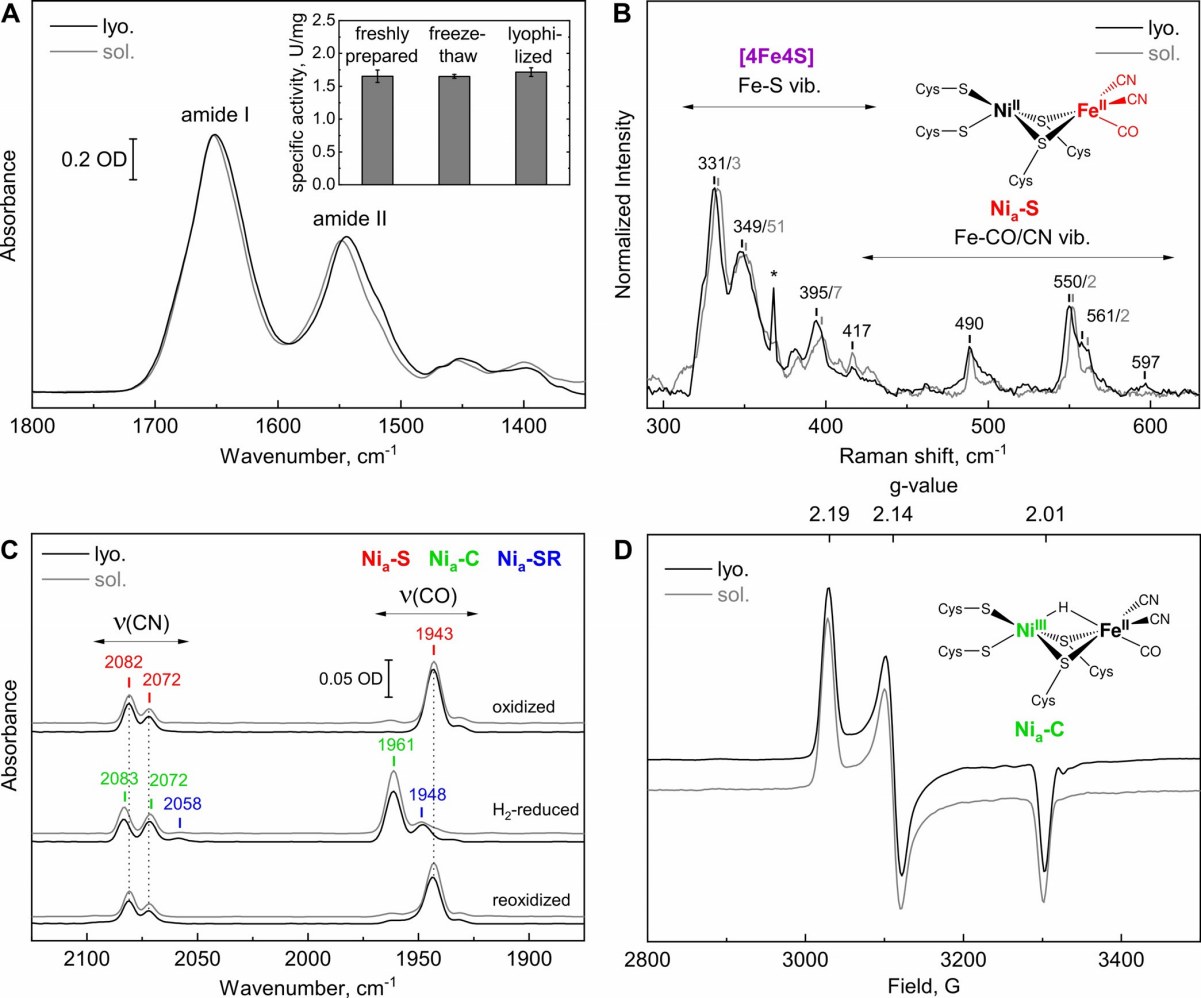
Spectroscopic and biochemical characterization of lyophilized (lyo.) *Re*RH compared to freshly prepared enzyme solution (sol.). A) IR spectroscopic signature reflecting the amide I and II vibrational modes ^[38]^ of the oxidized enzyme. The inset shows the specific H_2_‐oxidation activity of freshly prepared, freeze‐thawed and re‐dissolved lyophilized samples. B) RR spectra of the oxidized enzyme obtained with 458 nm excitation and normalized to the most intense active site signal at 550–552 cm^−1^. The small shifts of 1–2 cm^−1^ likely reflect differences between protein purified from cells grown with either ^57^Fe (lyo.) or iron with natural isotope distribution (sol.). The sharp signal marked with an asterisk derives from an optical artefact of the Raman spectrometer. C) IR spectra of *Re*RH, oxidized (top), H_2_‐reduced (middle) and subsequently re‐oxidized with air (bottom). For details about the assigned active‐site redox states, see Figure S1 and Table S1. D) EPR spectra of the H_2_‐reduced enzyme in solution and as compressed lyophilizate (both measured at 80 K and with 1 mW microwave power). IR and EPR spectra of the protein solution are normalized to the intensity of the lyophilized sample.

The experimental setup introduced here is designed for the preparation of well‐defined redox states of metalloenzymes and their concomitant multi‐spectroscopic analysis under various experimental conditions and in different sample forms. The sample compartment consists of a gas‐tight chamber connected to an external vacuum pump and a variety of dry or humidified gases (Figures 2 and S2). The temperature can be set between 80 K and 333 K. Our newly developed approach also allows the in situ analysis of protein solutions (Figures S3 and S11), lyophilizates (Figure 3 C) and single crystals (Figures 5 and S10) by IR transmission spectroscopy. Since the whole sample compartment is portable and gas‐tight, it can also be disconnected from the remaining setup and moved (Figure S2), for example, for RR spectroscopic analysis of the same sample or further sample treatment in an anaerobic chamber.

To illustrate the capabilities of our platform, we first describe the preparation and spectroscopic characterization of highly concentrated lyophilized samples of *Re*RH, suitable for conducting NRVS experiments. Upon incubation with H_2_, *Re*RH resides predominantly in the so‐called Ni_a_‐C catalytic intermediate. This species harbors a bridging hydride between the Fe^II^ and Ni^III^ ions (Figure S1), which is of general interest in biological and chemical catalysis.[^33^, ^34^, ^35^] So far, experimental evidence for the presence of the hydride in this state has only been provided by EPR spectroscopy.[^35^, ^36^, ^37^] Currently, the only vibrational spectroscopic method suitable to monitor metal−ligand bonding in this light‐sensitive species (Figure S4) is NRVS.[^19^, ^20^] Observation of active‐site metal−ligand vibrations with this technique, however, typically requires protein concentrations above 1 mm,[^27^, ^28^] which is the upper concentration limit for purified *Re*RH in solution. To overcome this limitation, we used our setup for a gentle lyophilization to prepare highly concentrated enzyme samples while preserving their catalytic activity. NRVS was performed subsequently in combination with density functional theory (DFT) calculations to reveal structural details of the Ni_a_‐C state.

In order to obtain highly concentrated samples in the Ni_a_‐C redox state, 0.5 mm protein solution of *Re*RH was flash‐frozen at 77 K and transferred to the sample compartment (Figures 2 A and S2). Subsequently, water was removed at controlled temperature and pressure by applying a mild vacuum of 0.1 mbar while slowly warming up the sample to 243 K. After completion of the lyophilization, the sample compartment was flushed with dry H_2_ gas to reduce the enzyme. Finally, compression of the protein lyophilizate, using a spatula and a pestle, yielded highly dense samples (equivalent to 4–5 mm, see Supporting Information for details) suitable for demanding methods such as NRVS (Figure 2 C). For further details, see the Materials and Methods section in the Supporting Information and Figure S2.

To verify structural integrity and functionality of the lyophilized oxidized and H_2_‐reduced enzyme, we measured the H_2_‐oxidation activity and applied EPR, IR and RR spectroscopy (Figure 3). This multi‐spectroscopic approach was only applicable due to the design of the setup. The IR signature of the amide I and amide II vibrational modes is related to the absorption of the (polyamide) backbone of the protein and therefore reflects its secondary and tertiary structure.^[38]^ The similarity between the spectra of the lyophilized and solution‐phase samples indicates that the freeze‐drying process did not affect the integrity of the protein structure (Figure 3 A). Additionally, lyophilized and subsequently re‐dissolved *Re*RH showed the same hydrogenase activity as freshly isolated enzyme, indicating that the metal cofactors and amino acid side chains responsible for substrate conversion and proton/electron transfer were not altered by lyophilization (Figure 3 A inset). The integrity of the iron–sulfur cluster relay was verified by almost identical Fe−S signals in the corresponding RR spectra (Figure 3 B). Likewise, metal−ligand vibrations, characteristic for the most oxidized, hydrogen‐binding [NiFe] intermediate, termed Ni_a_‐S (Figure S1), were observed, thereby confirming a native active‐site structure of oxidized *Re*RH lyophilizate (Figure 3 B).^[20]^

Intriguingly, the lyophilized enzyme still reacts with molecular hydrogen, as monitored by in situ IR spectroscopy (Figure 3 C). The biologically unusual CO and CN^−^ ligands at the active site of [NiFe]‐hydrogenases give rise to valuable IR marker bands that allow the identification of individual redox states of the [NiFe] center.[^39^, ^40^, ^41^] The H_2_‐dependent formation of the Ni_a_‐C state indicates a redox‐active *Re*RH in the compressed and H_2_‐reduced lyophilizate. Complementary EPR spectra display the typical rhombic signature of the Ni_a_‐C state,^[35]^ confirming a native active‐site structure after lyophilization, reduction and compression (Figure 3 D).

**Figure 4:**
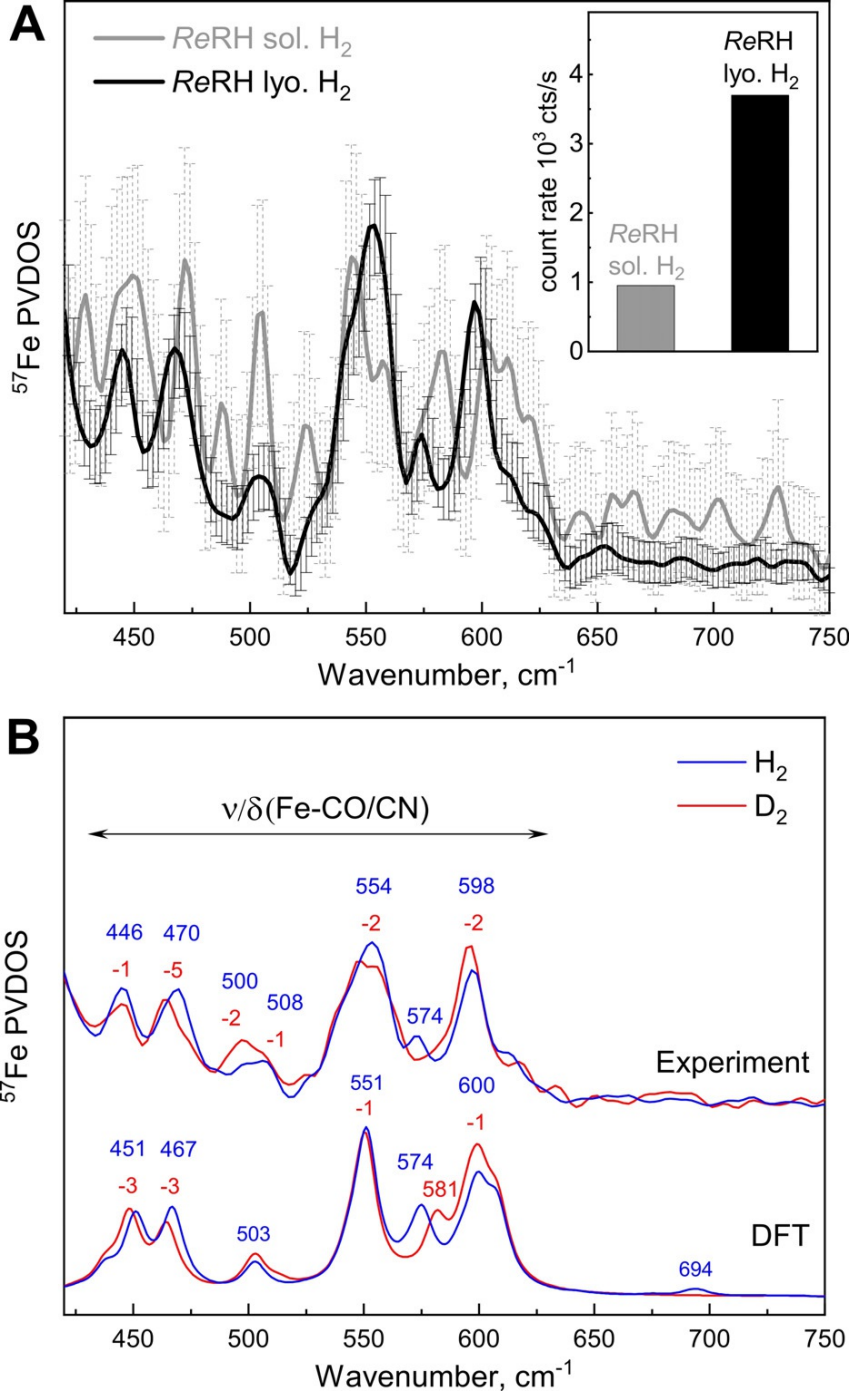
Nuclear resonance vibrational spectra of *Re*RH in the Ni_a_‐C state. A) NRVS data of lyophilized, H_2_‐reduced and compressed *Re*RH (lyo., black trace) compared to the corresponding protein solution at 0.5 mm (sol., gray trace). Both spectra were recorded for 12 hours. The inset displays the corresponding count rates at the elastic peak. B) Experimental NRVS data of lyophilized *Re*RH (top) incubated with H_2_ (blue traces, 26 hours accumulation) or D_2_ (red traces, 16 hours accumulation) in comparison to the corresponding DFT‐calculated ^57^Fe‐PVDOS spectra, which were obtained using the model shown in Figure S6. NRVS data including error bars are displayed in Figure S9.

The lyophilization procedure described above yielded highly concentrated, ^57^Fe‐enriched *Re*RH samples, which allowed NRVS‐based detection of active‐site vibrations of the Ni_a_‐C state. The NRVS data shown in Figure 4 comprise the spectral regions characteristic for Fe−CO/CN modes (400–650 cm^−1^) and Ni−H−Fe wagging vibrations (650–800 cm^−1^).[^25^, ^26^, ^27^, ^28^] The count rate of the elastic peak accumulated by the detector increased by a factor of four for the lyophilized sample in comparison to the solution‐phase sample (Figure 4 A, inset), providing a significant improvement of the signal‐to‐noise ratio. Consequently, the error bars of the two sample spectra vary, on average, by a factor of four (Figure 4 A, see Supporting Information for details).

In order to specifically probe the metal‐bound hydride of the Ni_a_‐C state, lyophilized *Re*RH samples were treated with both H_2_ and D_2_ (Figure 4 B). The resulting experimental spectra were compared to simulated data obtained by DFT calculations (Figure 4 B), based on an *Re*RH active‐site homology model[^42^, ^43^] (Figures S5 and S6; for details, see the DFT Methods section of the Supporting Information). Analysis of the Ni_a_‐C spectrum obtained after incubation of *Re*RH with H_2_ (Figure 4 B, top blue trace) revealed two intense bands at 554 and 598 cm^−1^ and a weaker one at 574 cm^−1^. Additional spectral features appear at 446, 470, 500 and 508 cm^−1^. Previously, [NiFe]‐hydrogenase vibrational signals at these energies were attributed to normal modes dominated by Fe−CO and Fe−CN stretching and bending motions.[^19^, ^20^, ^21^, ^25^, ^26^, ^27^, ^28^] Overall, the agreement between the experimental and calculated spectra is good (Figure S7). The isolated Ni−H−Fe wagging motions are predicted to produce only very weak ^57^Fe‐PVDOS intensities in the 670–740 cm^−1^ region. The most noticeable spectral feature of this type is expected at 694 cm^−1^ and formed by two modes at 692 and 696 cm^−1^, each characterized by approximately 30 % μH‐PVDOS but only subtle 1 % ^57^Fe‐PVDOS. A discrete hydride band could not be resolved by NRVS due to the low signal‐to‐noise ratio in this spectral region, far away from the elastic Mössbauer peak. However, a distinct H/D‐sensitive band observed at 574 cm^−1^ is predicted to arise from two modes calculated at 574 and 576 cm^−1^ (7 % ^57^Fe‐PVDOS each), both of which are combinations of μH wagging and C−NH_2_ bending of the nearby Arg411 guanidium group (Figures S7A and S8). The NRVS data recorded from *Re*RH incubated with D_2_ (Figure 4 B, top red trace) reveal small shifts of 1–5 cm^−1^ to lower frequencies for several bands. This can be explained by deuteride motions contributing to the Fe−CO(CN)‐dominated modes,[^20^, ^44^, ^45^] with a μD‐PVDOS maximum (23 %) calculated at 501 cm^−1^ for Ni_a_‐C(μD) (Figures S7B and S8, normal mode animations provided in Supporting Information). Most prominently, the 574 cm^−1^ band detected for the H_2_‐reduced sample is absent in spectra from both the experimental D_2_‐treated sample and the calculated Ni_a_‐C(μD) model. Moreover, a newly emerging signal (calculated Ni_a_‐C(μD) mode at 581 cm^−1^) can be observed as a weak shoulder at ca. 586 cm^−1^, close to the nearby high‐intensity feature centered at 596 cm^−1^, indicating the presence of the μD deuteride. In summary, application of the novel setup proved to be beneficial for the enrichment of *Re*RH, thereby enabling NRVS characterization of the reduced enzyme. Rationalized by DFT calculations, these data allowed insights into active‐site metal−ligand vibrations including normal modes that reflect hydride coordination in the Ni_a_‐C redox state.

**Figure 5:**
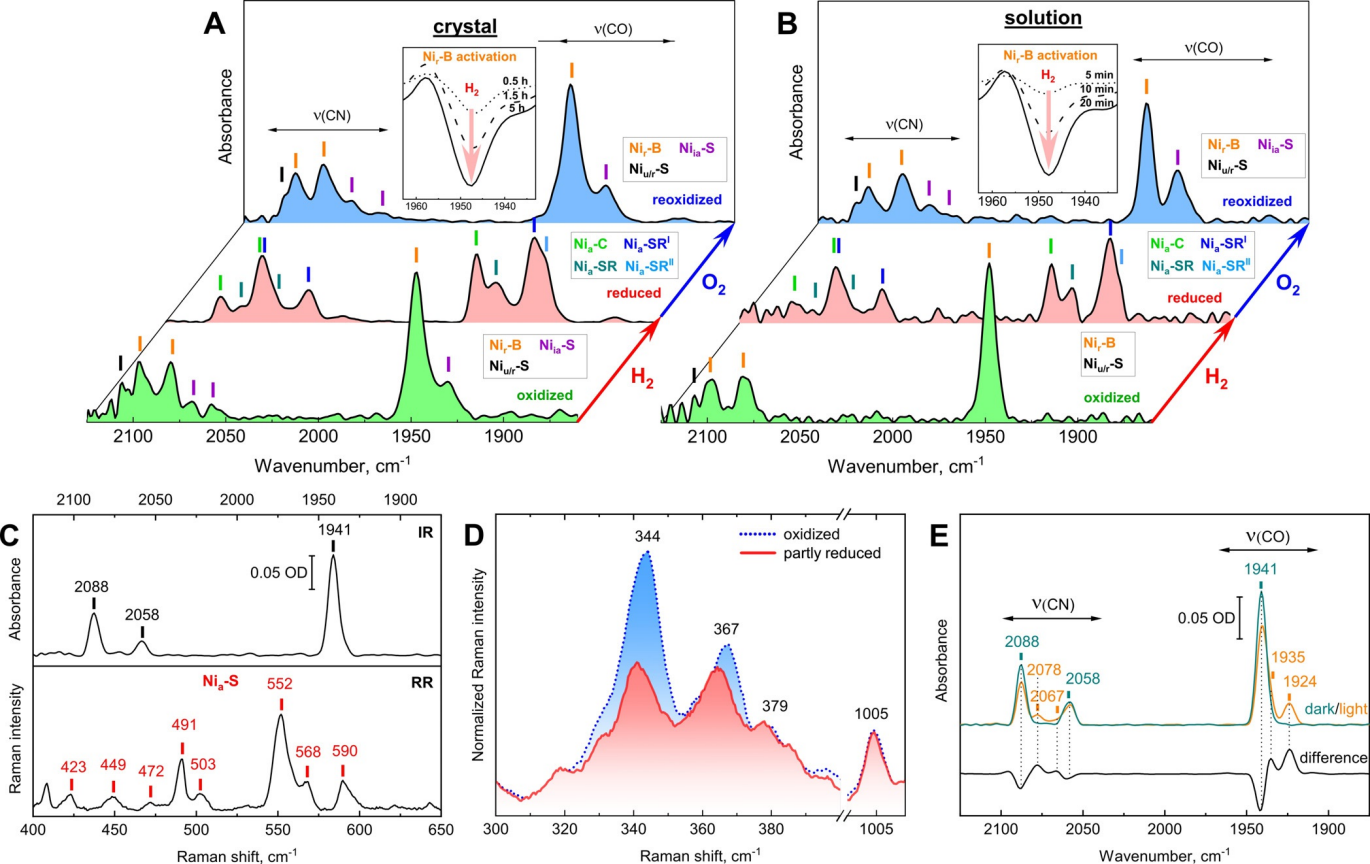
IR and RR spectra of *Re*MBH in the crystal and solution phase. In situ IR spectra of the oxidized (green), reduced (red) and reoxidized (blue) state of A) an enzyme crystal and B) protein solution after consecutive exposure to various humidified gases (N_2_, H_2_, synthetic air) at 277 K. The insets show the reduced‐minus‐oxidized difference spectra after different times of H_2_ exposure. For details on the spectroscopic transformations, see Figure S11. Details regarding the redox states assigned to the active site are described in Figure S1 and Table S2. C) IR spectrum (top trace) of a protein crystal grown under reducing atmosphere (95 % N_2_ and 5 % H_2_) and the corresponding RR spectrum obtained with 568 nm laser excitation (bottom trace) of the same crystal, both measured at 80 K. D) RR spectra displaying the spectral region characteristic for iron–sulfur cluster vibrational modes of oxidized (blue trace) and partially reduced (red trace) *Re*MBH crystals (the latter was grown under an atmosphere of 5 % H_2_ and 95 % N_2_). Spectra were recorded at 80 K with 514 nm laser excitation. RR spectra are normalized to a non‐resonant band of the amino acid side chain of phenylalanine at 1005 cm^−1^. E) IR spectrum of a protein crystal grown under reducing atmosphere (95 % N_2_ and 5 % H_2_) before (dark, cyan) and after (light, orange) 8 hours illumination with the focused beam of a collimated 455 nm LED at 80 K. The corresponding light‐minus‐dark difference spectrum is shown in black.

Using *Re*MBH as a model metalloenzyme, we next demonstrate the applicability of our setup for the characterization of protein crystals by multiple spectroscopic techniques (Figures 2 B and S10). To obtain a comprehensive understanding of redox processes in [NiFe]‐hydrogenase crystals, we first compared in situ IR spectra of aerobically grown protein crystals and solution (Figure 5 A,B). Unlike Ash et al., who employed electrochemical control in combination with redox mediators to induce redox transitions within crystals of Hydrogenase 1 from *Escherichia coli*,^[46]^ our design allows reduction of the hydrogenase with its native substrate H_2_ and (re)oxidation with the inhibitor O_2_. Thus, our setup allows monitoring physiologically relevant redox processes at the [NiFe] active site, as exemplified for the reductive activation process of oxidized *Re*MBH in the following.

When exposed to oxidizing conditions, for example, upon incubation with air (Figure 5 A,B, green traces), O_2_‐tolerant hydrogenases like *Re*MBH typically switch to a non‐catalytic but easily reactivatable “resting” state, called Ni_r_‐B, which features a bridging hydroxide between Fe and Ni (Figures 5 A,B and S1).^[47]^ In principle, Ni_r_‐B may be directly activated by reaction of the [NiFe] site with H_2_, as suggested by Kurkin et al.^[48]^ Alternatively, activation could be accomplished indirectly by reverse, Fe−S cluster‐mediated transfer of H_2_‐derived electrons delivered by catalytically active hydrogenase molecules.^[49]^ Within crystals, however, each *Re*MBH heterodimer is locked at a fixed position and unable to rotate, thereby severely hampering electron transfer between different hydrogenase molecules. This would affect indirect but not direct reactivation. Thus, a comparison of solute and crystalline *Re*MBH samples provides a unique possibility to analyze H_2_‐mediated reactivation behavior of [NiFe]‐hydrogenase residing in the Ni_r_‐B state.

In fact, complete active‐site reduction in an *Re*MBH crystal required 5 h incubation with 100 % H_2_ (Figures 5 A, red trace and S11A), whereas complete reduction in solution was accomplished within 30 min (Figures 5 B, red trace and S11B). Strikingly, O_2_ (although larger than H_2_) re‐oxidized previously reduced *Re*MBH crystals (Figure 5 A, blue trace) to full extent within less than 5 min (Figure S11A), demonstrating that the slow, H_2_‐mediated reactivation of the Ni_r_‐B state in crystalline samples is not caused by rate‐limiting gas diffusion. We therefore propose that the fast activation of Ni_r_‐B—as observed in solution—occurs via the indirect route, involving electron transfer from other hydrogenase molecules. Slow activation—as observed in the crystal phase—could be ascribed to less efficient direct activation by H_2_ or long‐range intermolecular electron transfer^[50]^ between metal centers of (translationally and rotationally locked) *Re*MBH heterodimers (Figure S12). These data demonstrate how our setup allows studying redox processes of gas‐converting enzymes in the crystal phase.

The redox state and behavior of a metalloenzyme may not only depend on the sample phase (crystal vs. solute), but also on the temperature and the sequence of steps during sample preparation that lead to the final state (which may not reflect thermodynamic equilibrium). These aspects are typically inaccessible by crystallography, thus requiring additional insight from in situ spectroscopy (vide supra) performed under identical conditions and on the same set of crystals.[^22^, ^51^] As most crystallographic data are acquired at cryogenic temperatures, it is of utmost importance to perform the corresponding spectroscopic experiments both at cryogenic and ambient temperatures. This way, information from cryogenic crystal structures can be adequately related to ambient‐temperature and/or solution‐phase data, thereby revealing the functionality of the native protein. The low‐temperature configuration for IR and RR spectroscopy of our new setup allows to conduct such experiments.

As an example, we investigated *Re*MBH crystals grown under moderately reducing conditions of 5 % H_2_ and 95 % N_2_. In case of hydrogenases, H_2_ specifically acts as a reducing agent, thereby defining the redox status of the sample. As revealed by IR spectra recorded from several crystals at 80 K (Figures 5 C, top trace and S13A), the [NiFe] active site forms one dominant redox state, whose IR signature has not been reported for *Re*MBH so far. To obtain further insight into this previously unknown active‐site state, we varied both the temperature (up to 283 K, Figure S13B) and the gas atmosphere (100 % H_2_ or 20 % O_2_ / 80 % N_2_, Figure S13C) over the crystal in situ. Surprisingly, neither had a notable impact on the IR pattern of the *Re*MBH crystals. This is in sharp contrast to crystals grown under aerobic conditions, which are easily manipulatable by adding either of these gases (vide supra), indicating that the new redox state is inactive. Moreover, crystals grown under 100 % H_2_ revealed similar spectroscopic signatures as those grown under 5 % H_2_ and 95 % N_2_ (Figure S13B), indicating that, for so‐far unknown reasons, the continuous presence of H_2_ during crystal growth traps *Re*MBH in this state. Taking advantage of the transferable sample holder, we next recorded complementary RR spectra of the same crystal grown under 5 % H_2_ / 95 % N_2_ (Figure 5 C, bottom trace). Surprisingly, the corresponding spectra reveal the characteristic signature of the Ni_a_‐S state^[21]^ and partial reduction of the Fe−S clusters^[52]^ (Figure 5 D). While the latter observation suggests successful reduction of the protein by H_2_, the former either indicates that the IR‐detected redox state is indistinguishable from Ni_a_‐S or that Ni_a_‐S is formed in situ as a photoproduct, due to illumination by the intense Raman probe laser. To test this hypothesis, we illuminated the crystal for 8 hours at 80 K with the focused beam of a collimated 455 nm LED (Figure 5 E). Two new species emerged in the concomitantly recorded IR spectra, indicating that the IR‐detected “dark” state is light‐sensitive and not identical with the Raman‐probed Ni_a_‐S state. This finding offers the opportunity to study the activation mechanism of inhibited redox states in detail.[^53^, ^54^]

On the one hand, these observations demonstrate that the redox status of crystallized metalloenzymes may not necessarily represent the expected equilibrium state, which is highly relevant for the interpretation of crystallographic data. On the other hand, the identification of a new redox state in the crystal phase offers an interesting avenue for combined X‐ray diffraction and vibrational spectroscopic studies, as previously performed with the same setup on the F_420_‐reducing hydrogenase from *Methanosarcina barkeri* ^[22]^ and the [FeFe]‐hydrogenase from *Desulfovibrio desulfuricans*.^[51]^ In total, the results presented here demonstrate the importance of in situ spectroscopic studies on protein crystals and the wide applicability of our new experimental platform.

## Conclusion

We have developed an experimental setup for multi‐spectroscopic in situ studies on gas‐converting metalloenzymes. Using this approach, enzymes can be studied in the form of single crystals, in solution or as lyophilizate—at various temperatures and gas atmospheres.

Freeze‐drying and mechanical compression yields protein samples with concentrations that are unattainable for many sensitive metalloenzymes in solution. This strategy can be utilized for studying such targets with low‐sensitivity spectroscopic techniques that provide otherwise inaccessible information on the enzyme structure and mechanism. The feasibility of this approach has been demonstrated by probing the hydride‐containing Ni^III^ intermediate of [NiFe]‐hydrogenase (Ni_a_‐C) by nuclear resonance vibrational spectroscopy.

Another valuable application is the in situ spectroscopic analysis of metalloenzyme samples in the crystalline and solution phase under variable experimental conditions. This allows the preparation, identification and functional analysis of catalytic intermediates in protein crystals, thereby enabling a more reliable interpretation of crystallographic data. This is particularly important for enzymes that are sensitive towards crystallization conditions and structural species that cannot be readily identified by X‐ray diffraction, as demonstrated for [NiFe]‐hydrogenases. Moreover, such studies allow evaluating the impact of the sample form on the reactivity towards different gaseous substrates. In this respect, we have compared activation and inhibition kinetics of crystallized and solvated [NiFe]‐hydrogenase, indicating that fast H_2_‐mediated activation in solution is accomplished via an indirect electron transfer mechanism.

In summary, the obtained results highlight the potential of our multi‐spectroscopic approach for studying metalloenzymes or other metal‐containing chemical systems, e.g. heterogeneous catalysts, under tight experimental control, thereby allowing new insights into structure and function.

## Conflict of interest

The authors declare no conflict of interest.

## Supporting information

As a service to our authors and readers, this journal provides supporting information supplied by the authors. Such materials are peer reviewed and may be re‐organized for online delivery, but are not copy‐edited or typeset. Technical support issues arising from supporting information (other than missing files) should be addressed to the authors.

Supplementary

Supplementary

Supplementary
