# Supplementary figures and images for: Exploring Structure and Function of Redox Intermediates in [NiFe]‐Hydrogenases by an Advanced Experimental Approach for Solvated, Lyophilized and Crystallized Metalloenzymes
Source: Angew Chem Int Ed Engl. 2021 May 5;60(29):15854–62. doi: 10.1002/anie.202100451 (PMC8360142; doi:10.1002/anie.202100451)

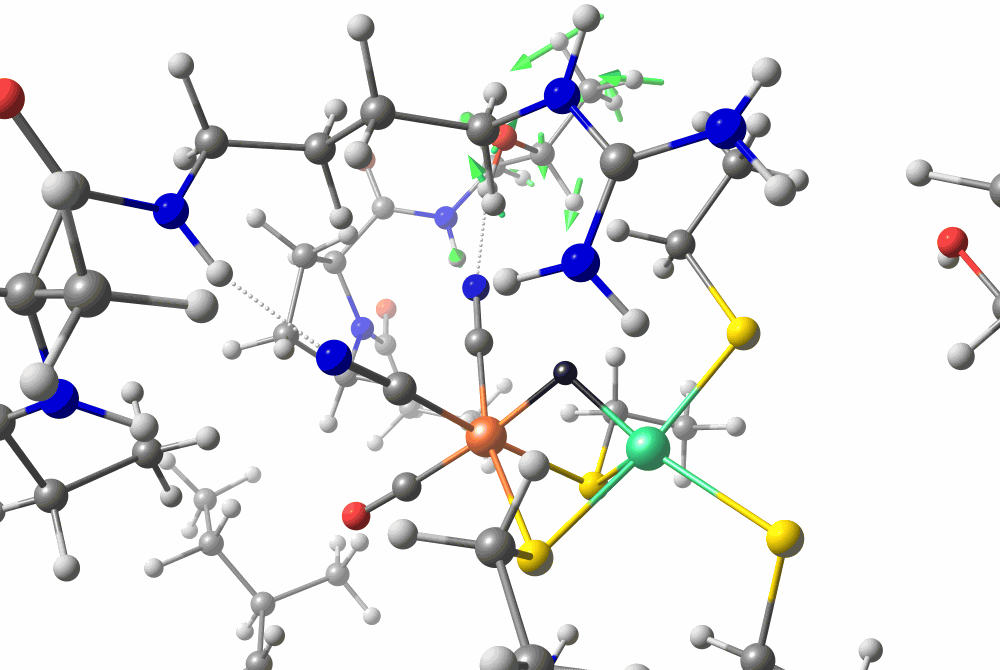

Supplement: Supplementary file 1 — Supplementary [file ANIE-60-15854-s001.zip › ReRH_Nia-C_muD_438cm-1.gif]

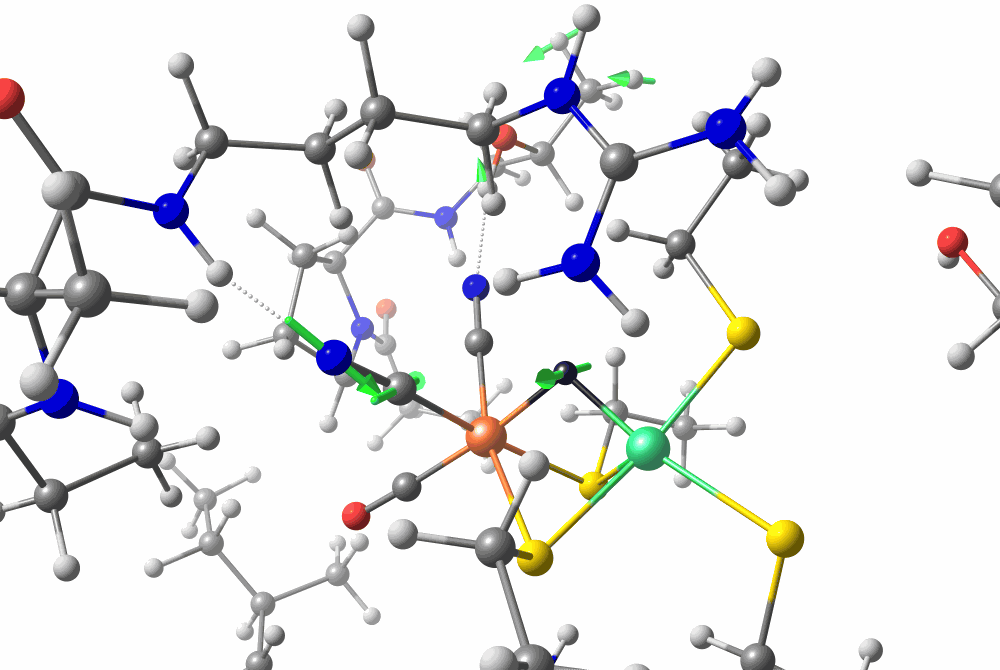

Supplement: Supplementary file 1 — Supplementary [file ANIE-60-15854-s001.zip › ReRH_Nia-C_muD_448cm-1.gif]

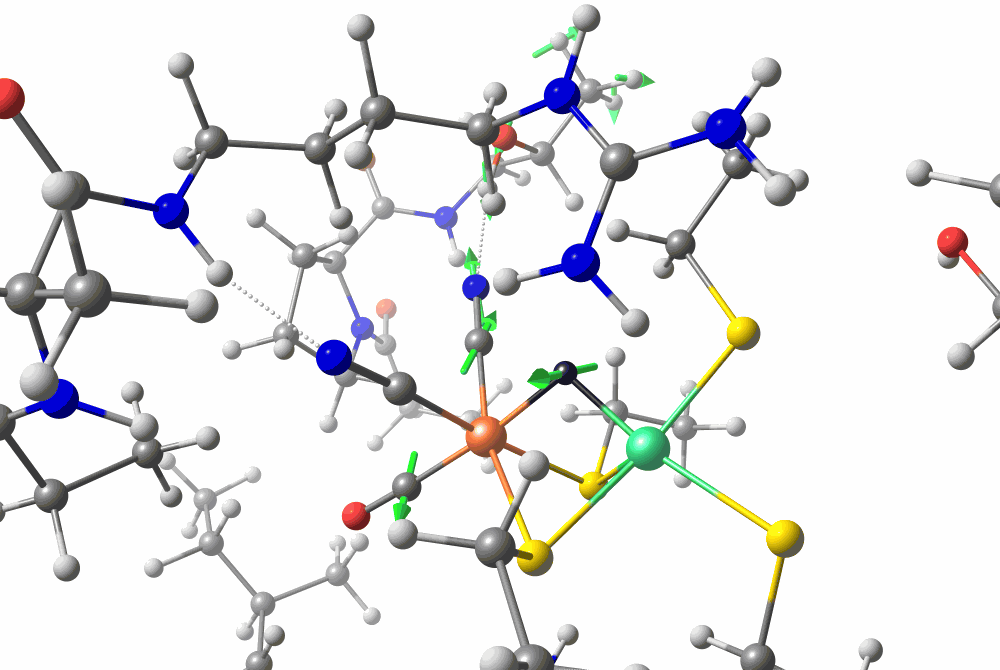

Supplement: Supplementary file 1 — Supplementary [file ANIE-60-15854-s001.zip › ReRH_Nia-C_muD_465cm-1.gif]

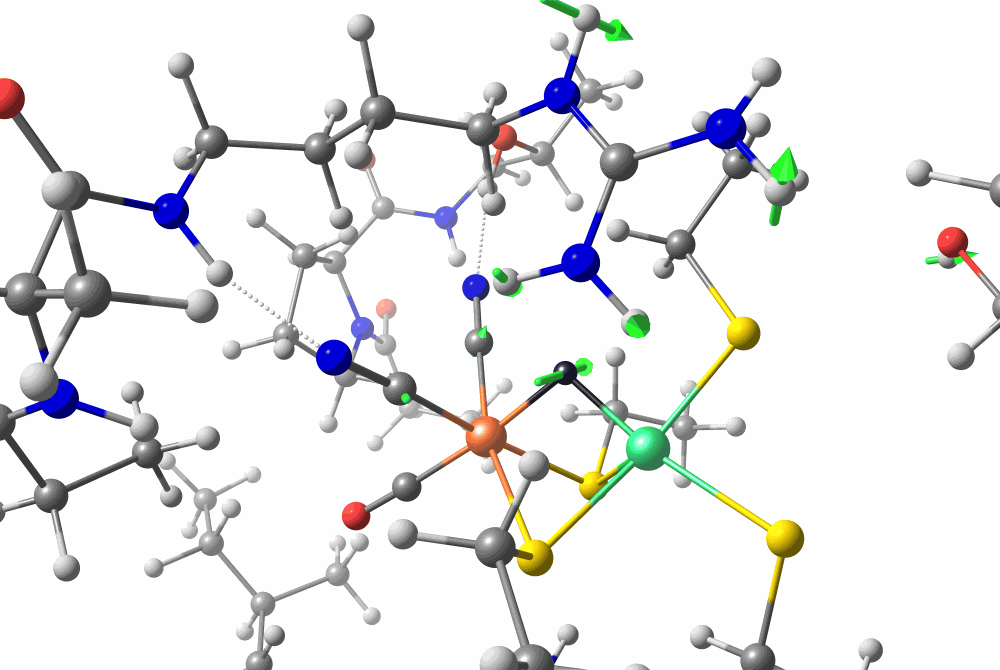

Supplement: Supplementary file 1 — Supplementary [file ANIE-60-15854-s001.zip › ReRH_Nia-C_muD_501cm-1.gif]

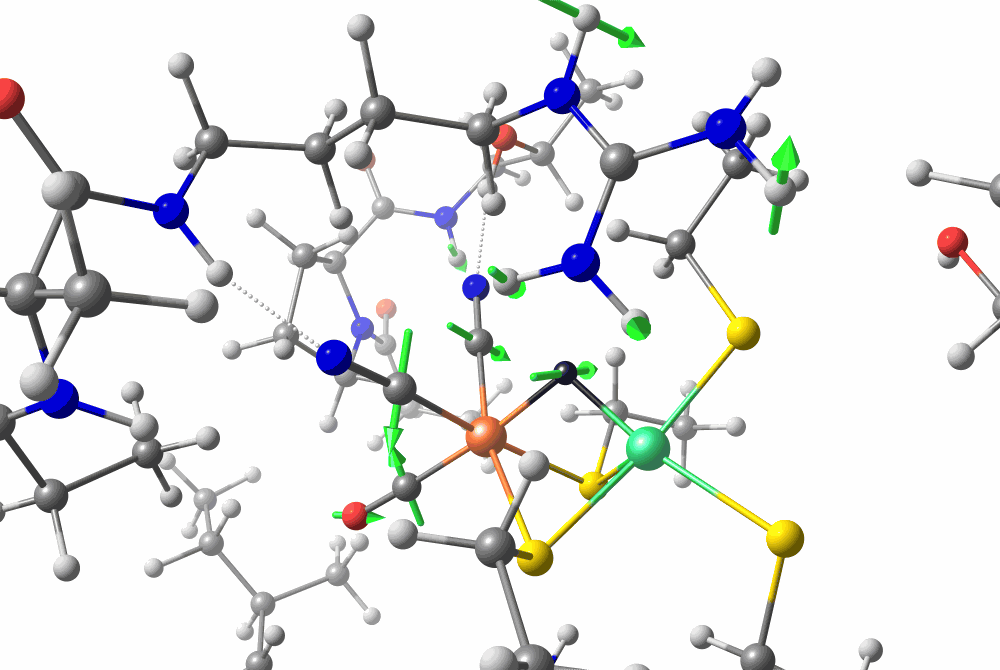

Supplement: Supplementary file 1 — Supplementary [file ANIE-60-15854-s001.zip › ReRH_Nia-C_muD_503cm-1.gif]

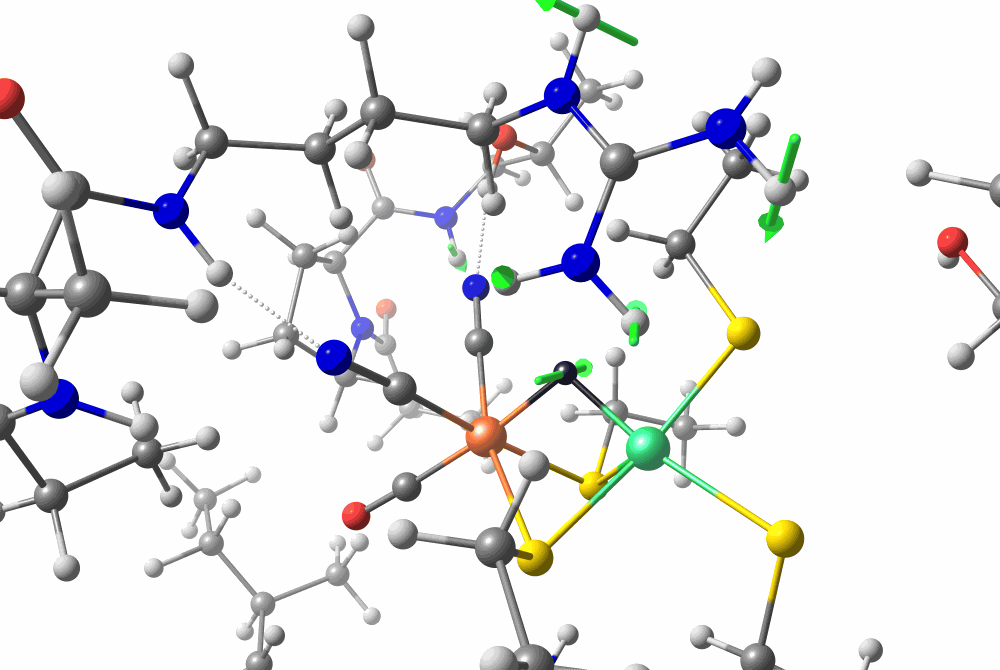

Supplement: Supplementary file 1 — Supplementary [file ANIE-60-15854-s001.zip › ReRH_Nia-C_muD_513cm-1.gif]

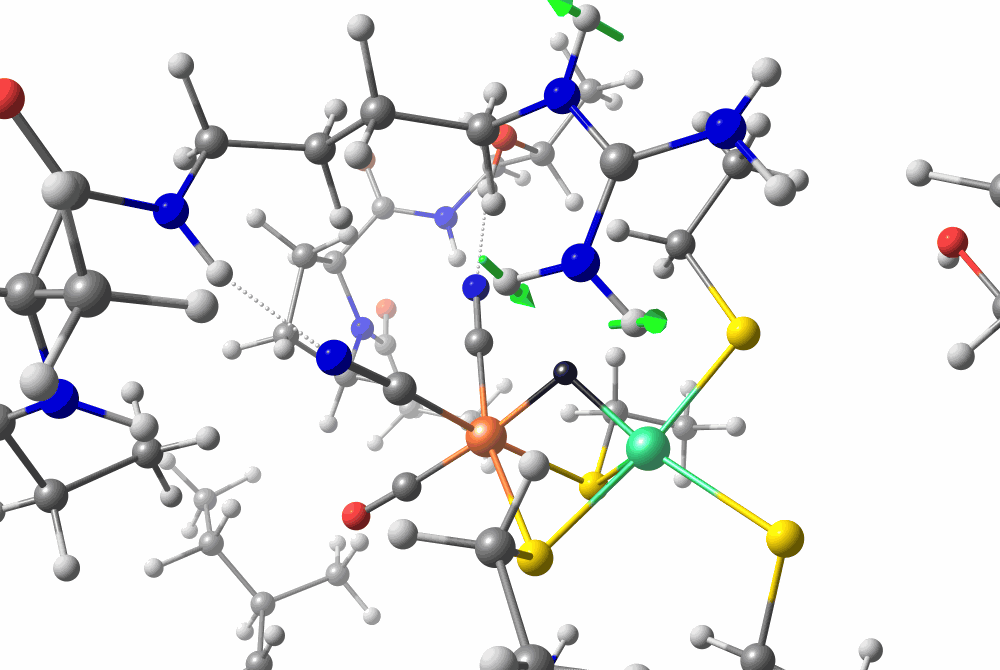

Supplement: Supplementary file 1 — Supplementary [file ANIE-60-15854-s001.zip › ReRH_Nia-C_muD_544cm-1.gif]

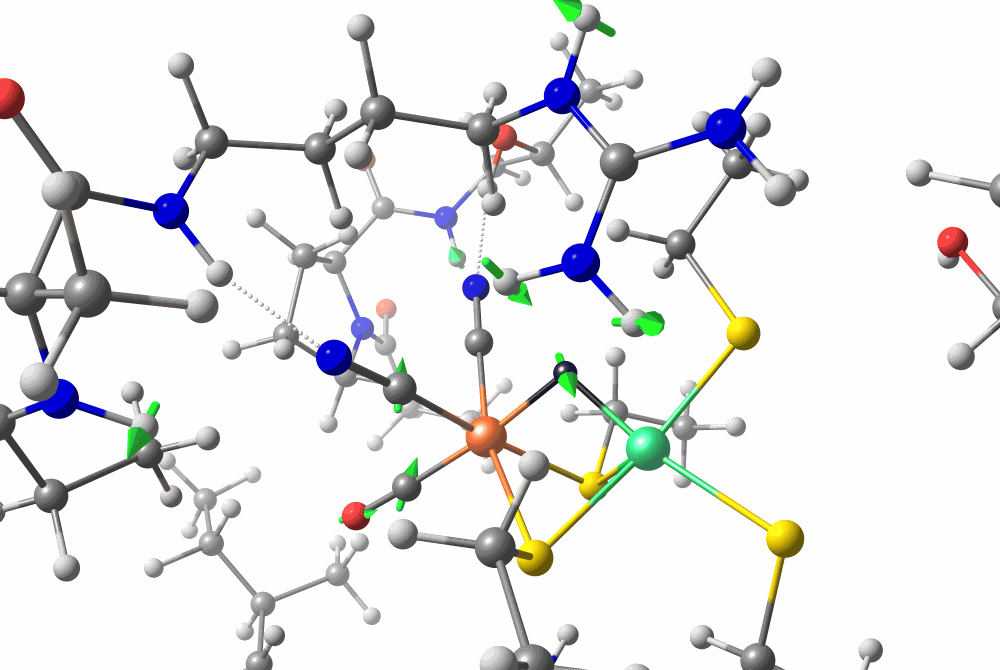

Supplement: Supplementary file 1 — Supplementary [file ANIE-60-15854-s001.zip › ReRH_Nia-C_muD_551cm-1.gif]

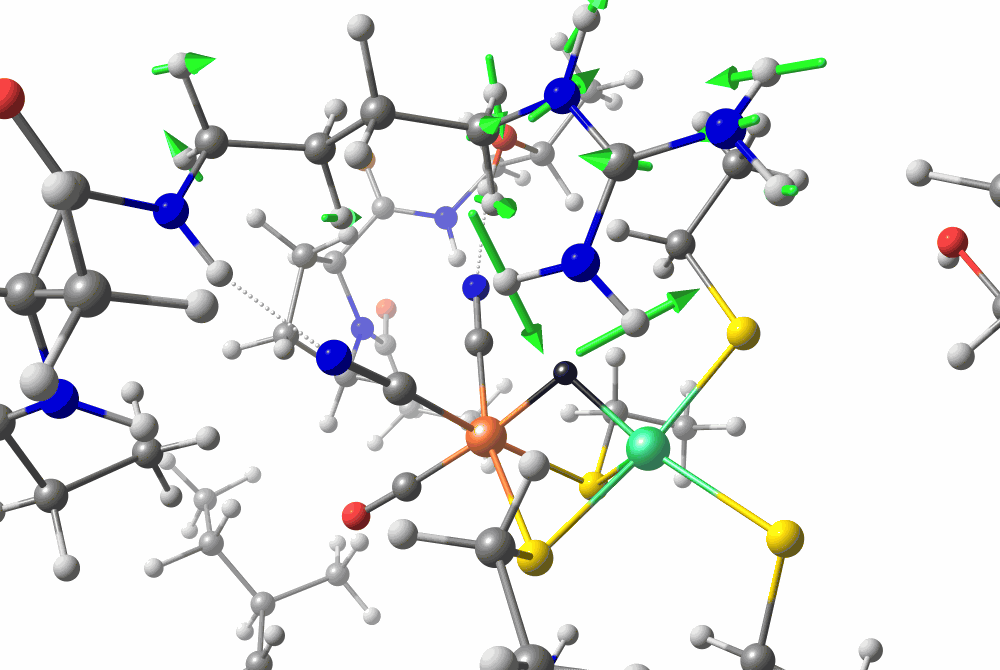

Supplement: Supplementary file 1 — Supplementary [file ANIE-60-15854-s001.zip › ReRH_Nia-C_muD_576cm-1.gif]

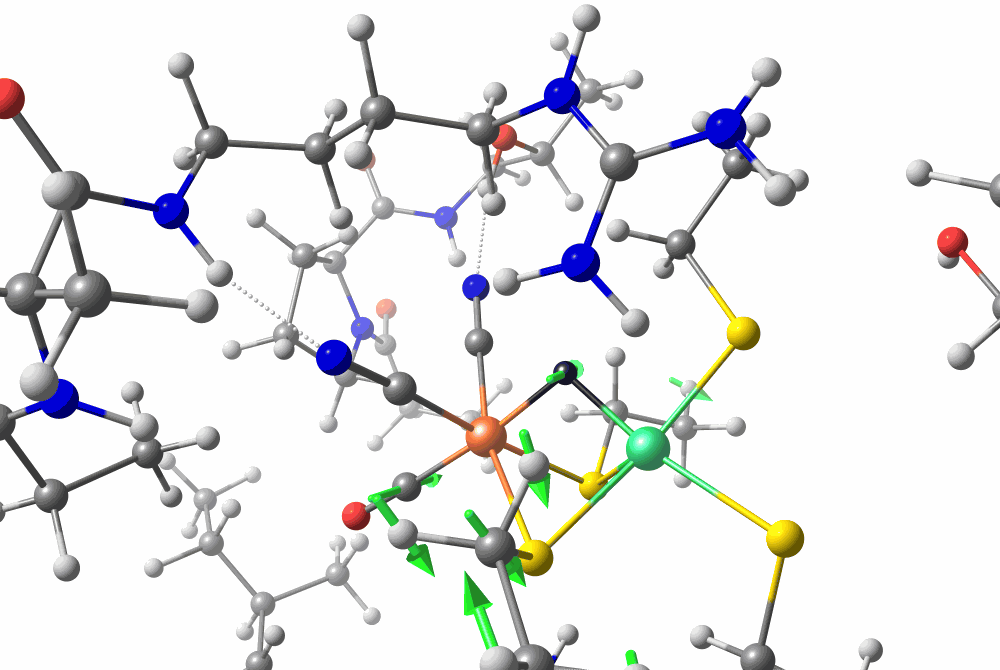

Supplement: Supplementary file 1 — Supplementary [file ANIE-60-15854-s001.zip › ReRH_Nia-C_muD_581cm-1.gif]

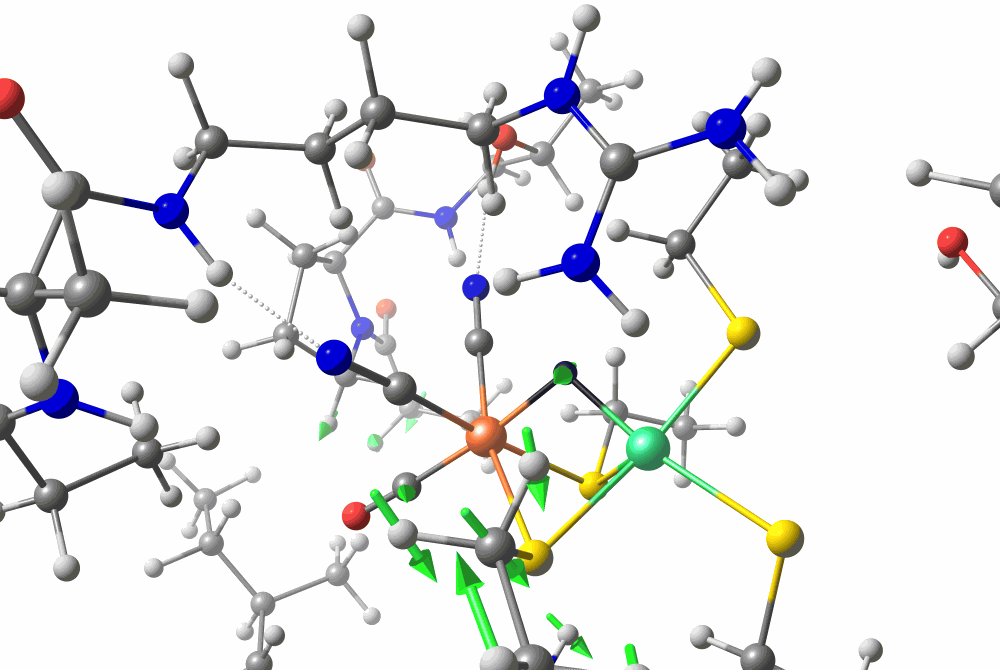

Supplement: Supplementary file 1 — Supplementary [file ANIE-60-15854-s001.zip › ReRH_Nia-C_muD_595cm-1.gif]

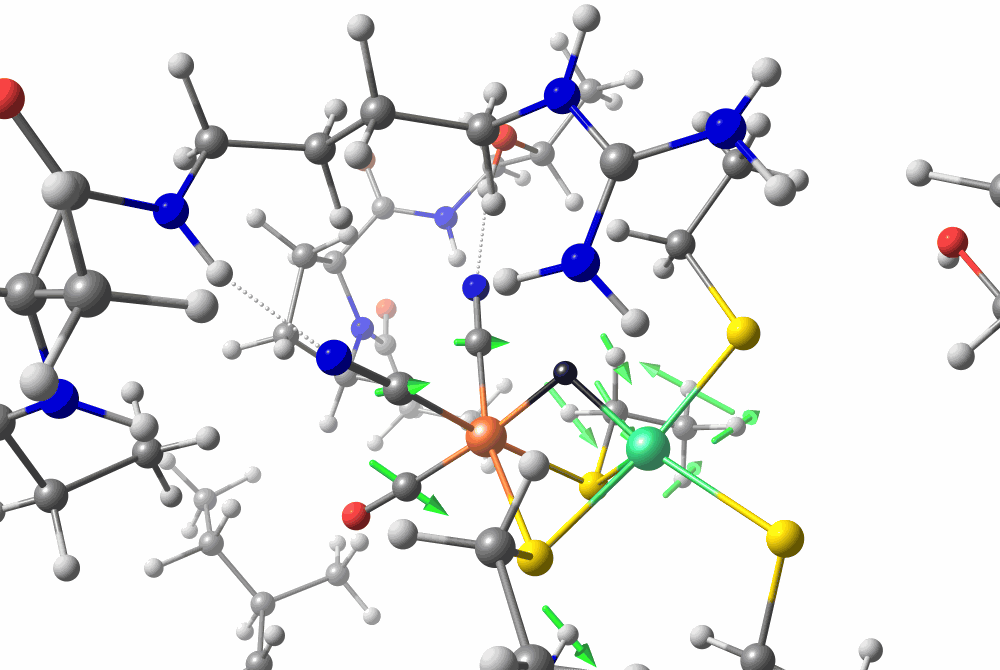

Supplement: Supplementary file 1 — Supplementary [file ANIE-60-15854-s001.zip › ReRH_Nia-C_muD_600cm-1.gif]

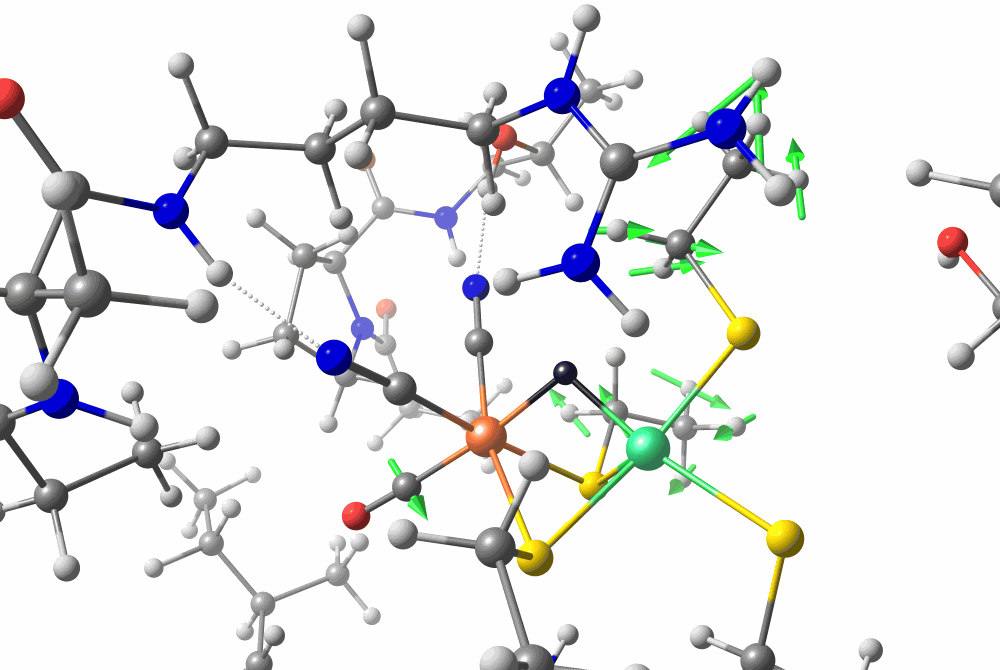

Supplement: Supplementary file 1 — Supplementary [file ANIE-60-15854-s001.zip › ReRH_Nia-C_muD_608cm-1.gif]

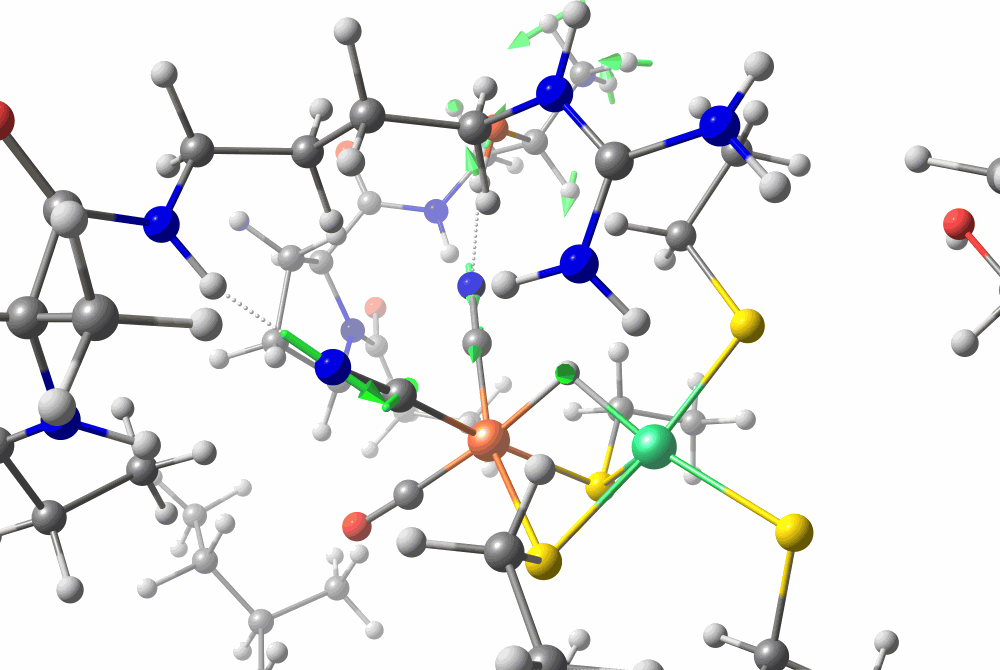

Supplement: Supplementary file 1 — Supplementary [file ANIE-60-15854-s001.zip › ReRH_Nia-C_muH_451cm-1.gif]

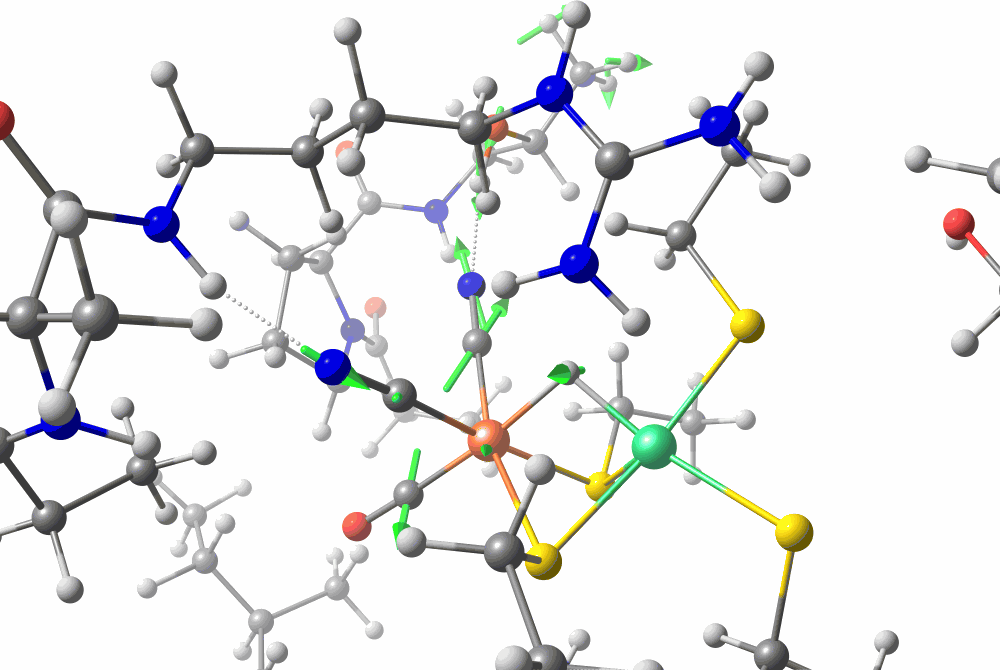

Supplement: Supplementary file 1 — Supplementary [file ANIE-60-15854-s001.zip › ReRH_Nia-C_muH_467cm-1.gif]

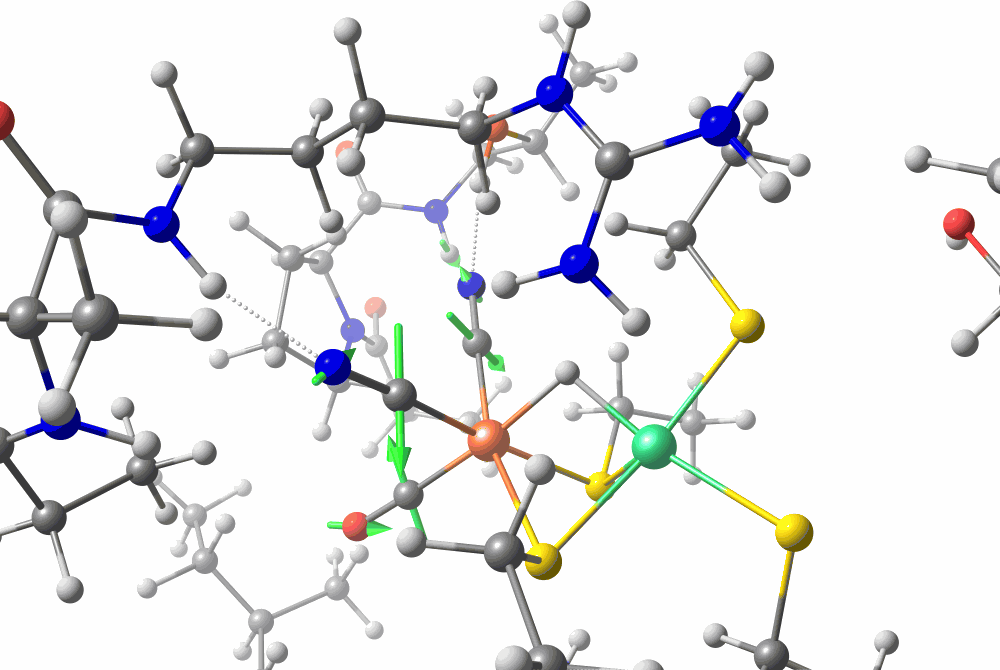

Supplement: Supplementary file 1 — Supplementary [file ANIE-60-15854-s001.zip › ReRH_Nia-C_muH_503cm-1.gif]

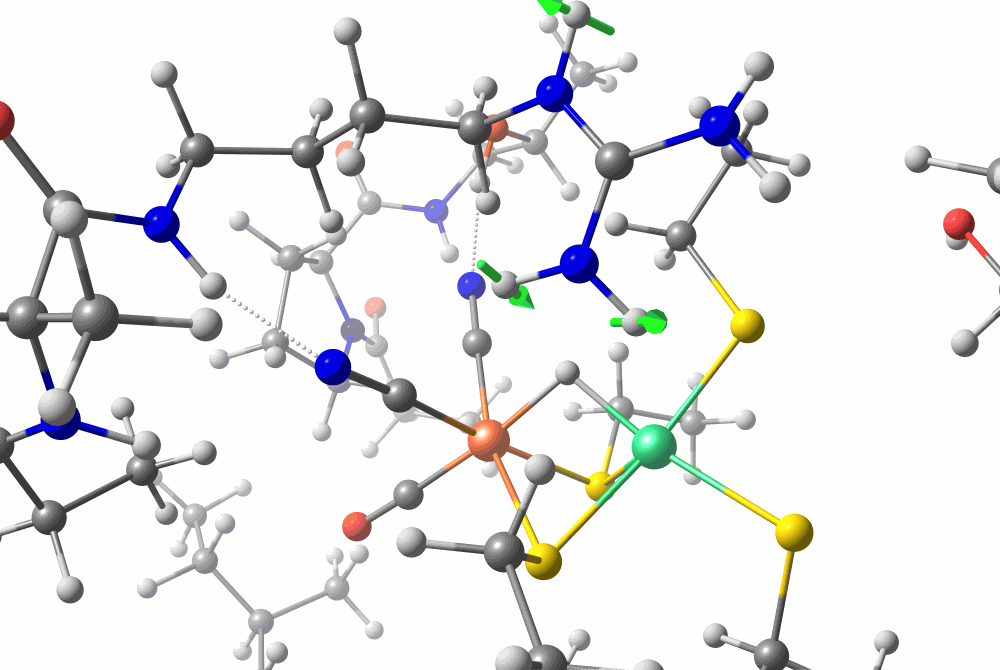

Supplement: Supplementary file 1 — Supplementary [file ANIE-60-15854-s001.zip › ReRH_Nia-C_muH_544cm-1.gif]

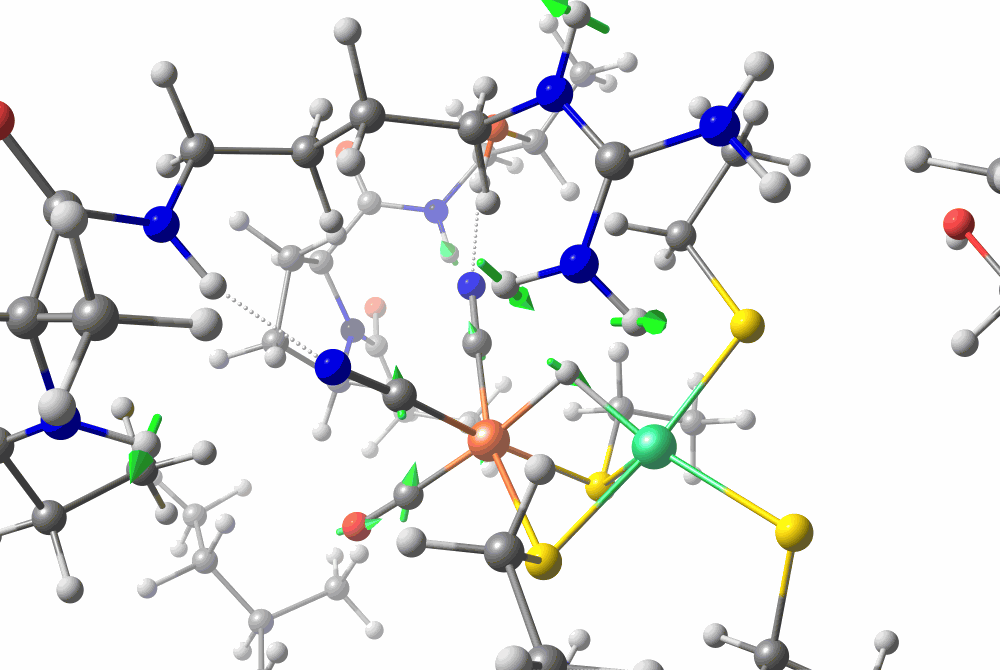

Supplement: Supplementary file 1 — Supplementary [file ANIE-60-15854-s001.zip › ReRH_Nia-C_muH_551cm-1.gif]

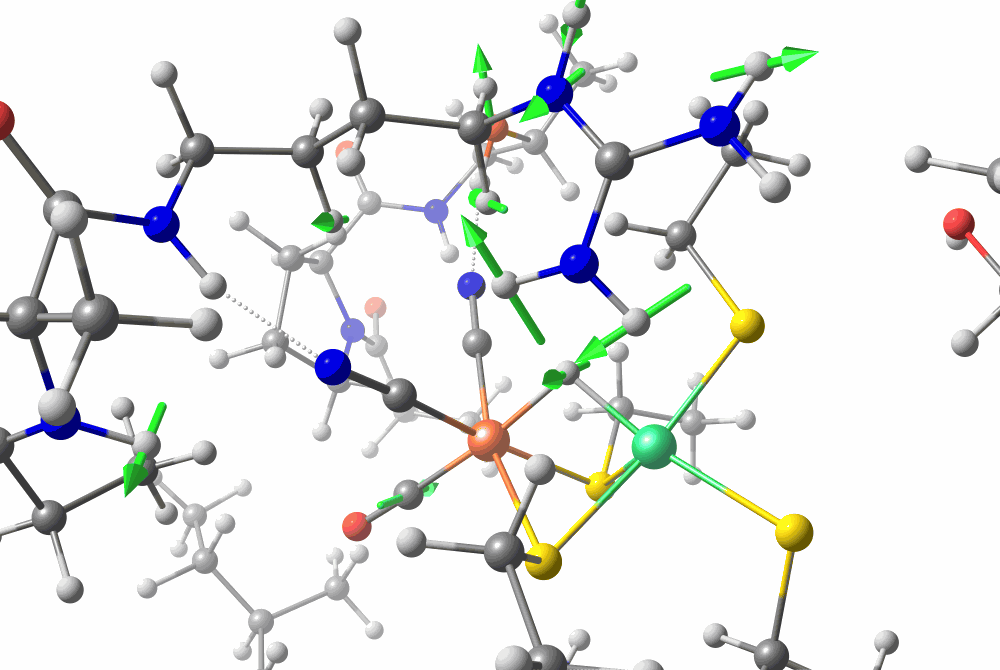

Supplement: Supplementary file 1 — Supplementary [file ANIE-60-15854-s001.zip › ReRH_Nia-C_muH_574cm-1.gif]

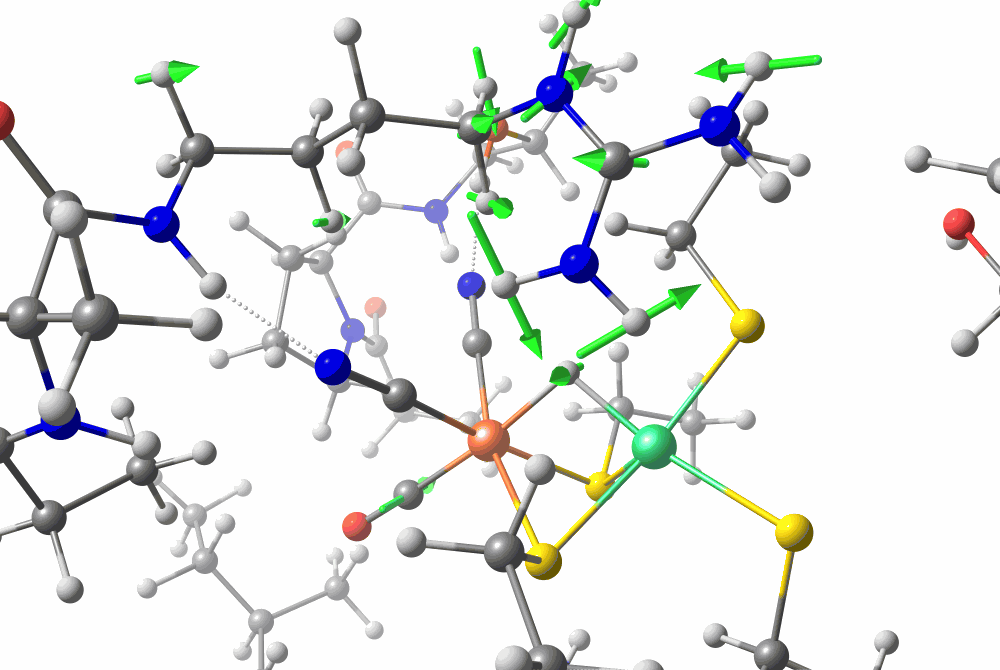

Supplement: Supplementary file 1 — Supplementary [file ANIE-60-15854-s001.zip › ReRH_Nia-C_muH_576cm-1.gif]

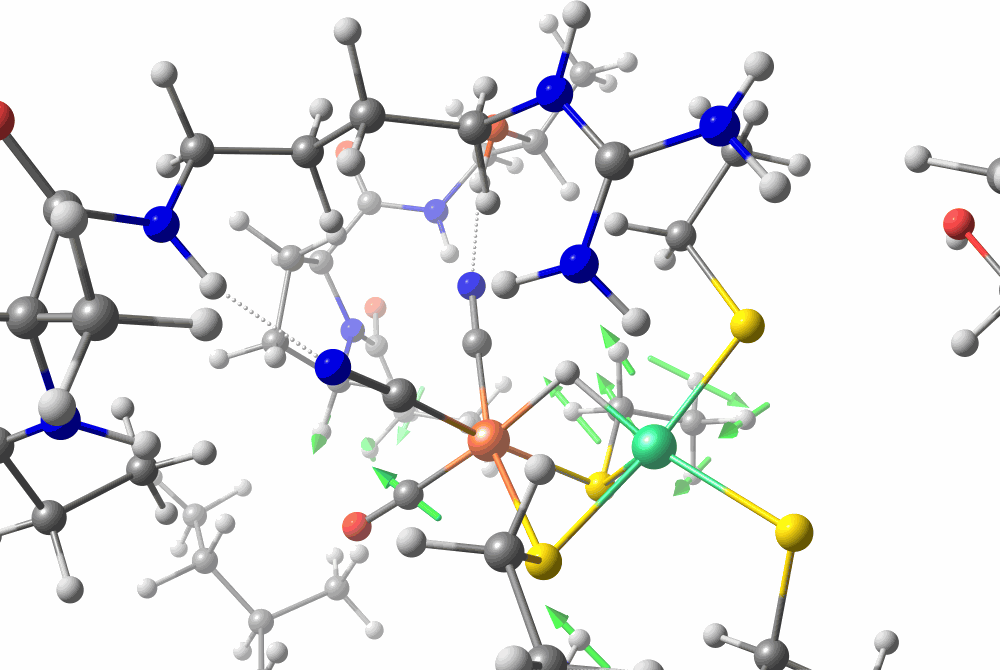

Supplement: Supplementary file 1 — Supplementary [file ANIE-60-15854-s001.zip › ReRH_Nia-C_muH_600cm-1.gif]

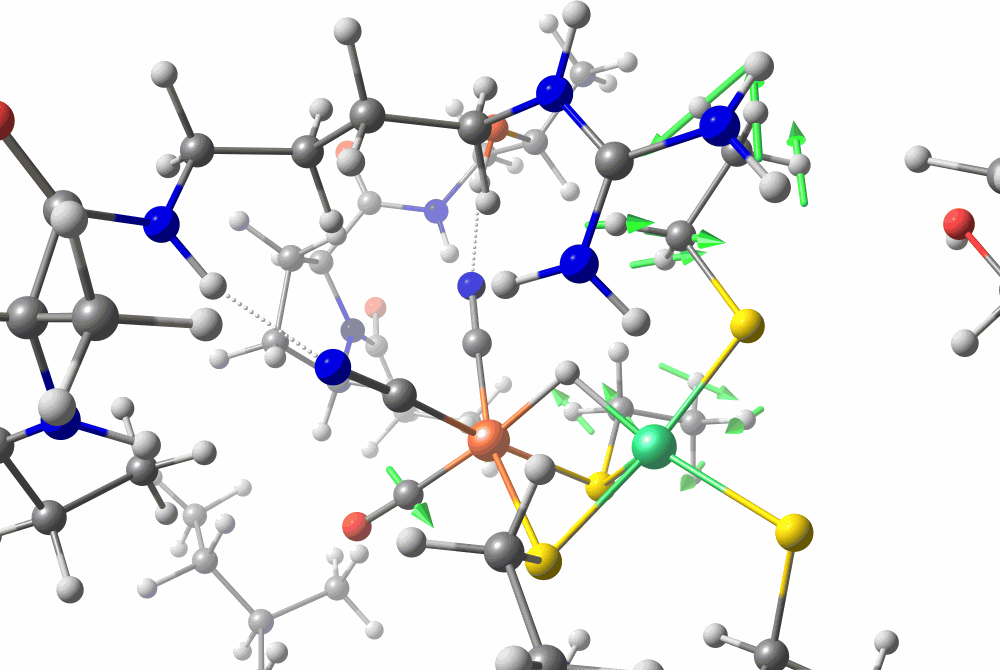

Supplement: Supplementary file 1 — Supplementary [file ANIE-60-15854-s001.zip › ReRH_Nia-C_muH_608cm-1.gif]

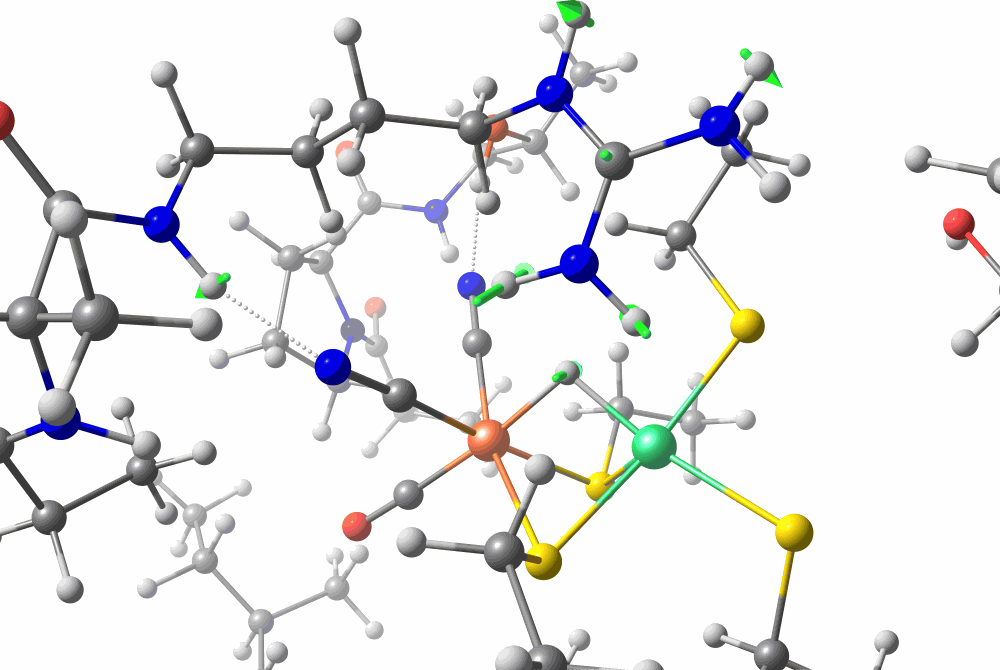

Supplement: Supplementary file 1 — Supplementary [file ANIE-60-15854-s001.zip › ReRH_Nia-C_muH_675cm-1.gif]

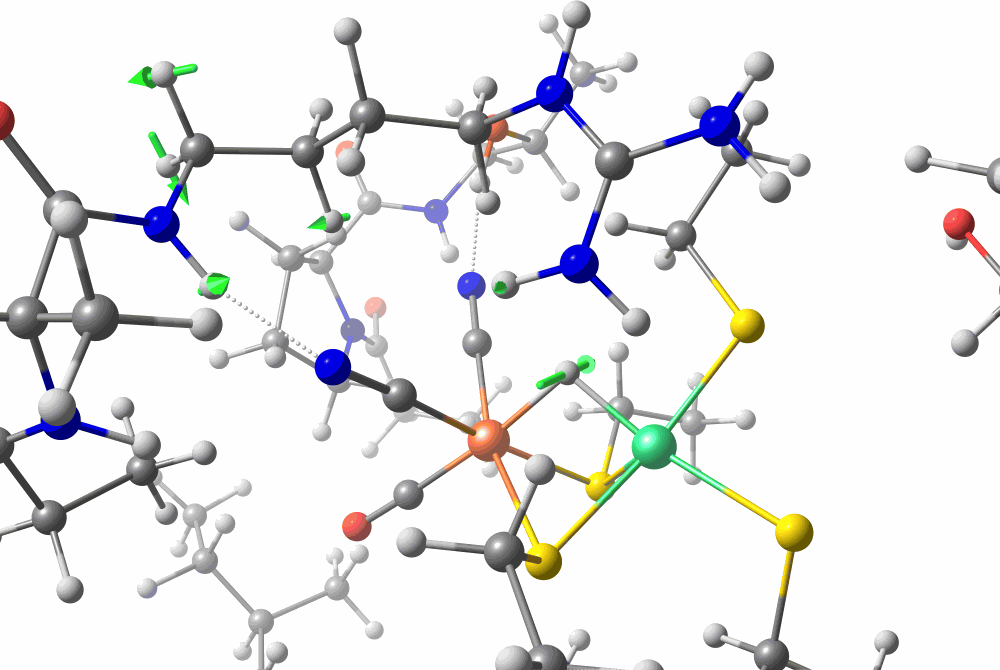

Supplement: Supplementary file 1 — Supplementary [file ANIE-60-15854-s001.zip › ReRH_Nia-C_muH_692cm-1.gif]

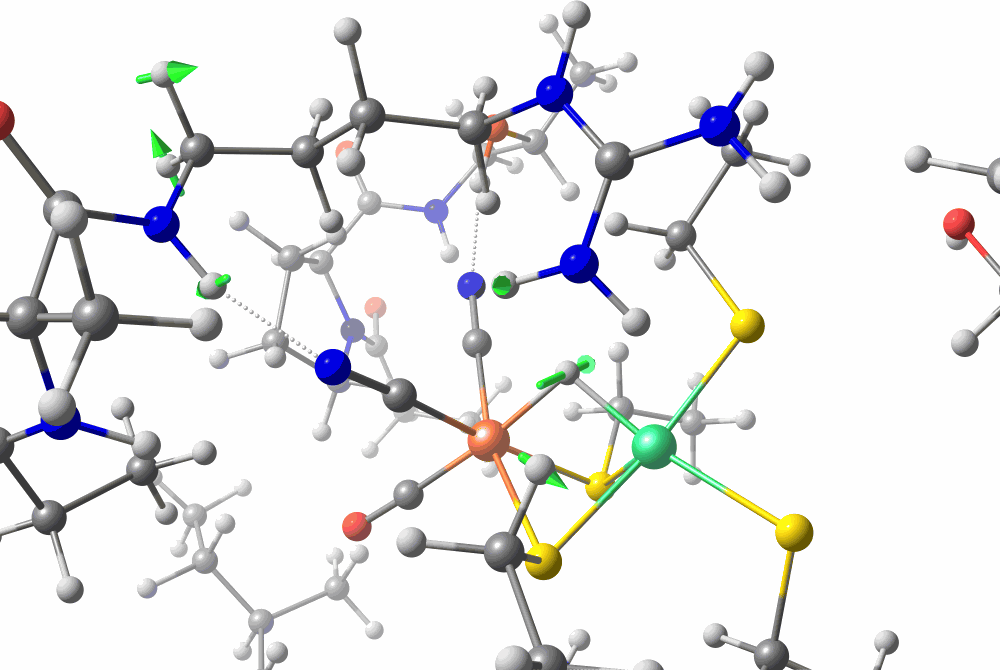

Supplement: Supplementary file 1 — Supplementary [file ANIE-60-15854-s001.zip › ReRH_Nia-C_muH_696cm-1.gif]

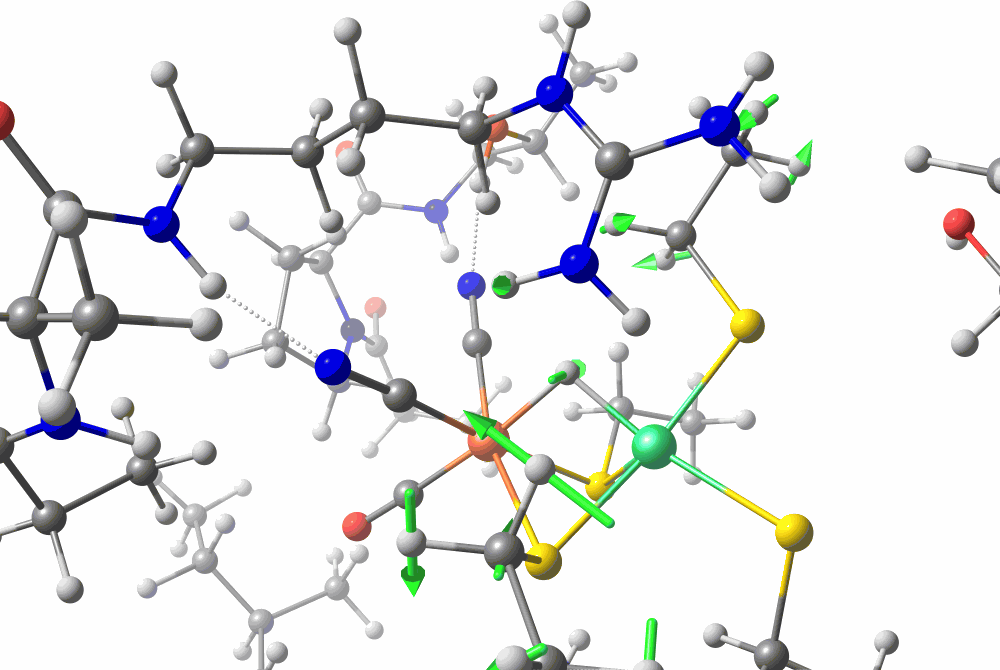

Supplement: Supplementary file 1 — Supplementary [file ANIE-60-15854-s001.zip › ReRH_Nia-C_muH_721cm-1.gif]

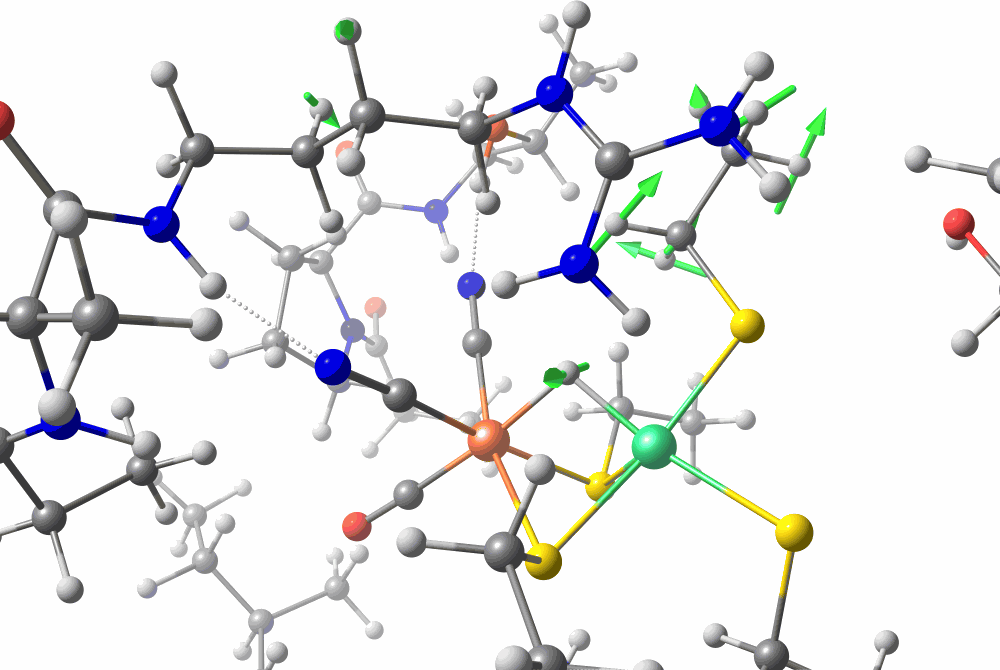

Supplement: Supplementary file 1 — Supplementary [file ANIE-60-15854-s001.zip › ReRH_Nia-C_muH_739cm-1.gif]
