# Supplementary material for: Exploring Structure and Function of Redox Intermediates in [NiFe]‐Hydrogenases by an Advanced Experimental Approach for Solvated, Lyophilized and Crystallized Metalloenzymes
Source: Angew Chem Int Ed Engl. 2021 May 5;60(29):15854–62. doi: 10.1002/anie.202100451 (PMC8360142; doi:10.1002/anie.202100451)
Supplement: Supplementary file 2 — Supplementary [file ANIE-60-15854-s003.pdf]

Angewandte Chemie
Eine Zeitschrift der Gesellschaft Deutscher Chemiker

Supporting Information

Exploring Structure and Function of Redox Intermediates in [NiFe]-Hydrogenases by an Advanced Experimental Approach for Solvated, Lyophilized and Crystallized Metalloenzymes

Christian Lorent, Vladimir Pelmenschikov, Stefan Frielingsdorf, Janna Schoknecht, Giorgio Caserta, Yoshitaka Yoda, Hongxin Wang, Kenji Tamasaku, Oliver Lenz, Stephen P. Cramer, Marius Horch,* Lars Lauterbach,* and Ingo Zebger**

anie_202100451_sm_miscellaneous_information.pdf
anie_202100451_sm_gif.zip
anie_202100451_sm_miscellaneous_information.xyz

Author Contributions

C.L. Conceptualization: Lead; Data curation: Lead; Formal analysis: Lead; Investigation: Lead; Methodology: Lead; Project administration: Lead; Validation: Lead; Visualization: Lead; Writing – original draft: Lead; Writing – review & editing: Lead
V.P. Data curation: Supporting; Formal analysis: Supporting; Investigation: Supporting; Methodology: Supporting; Validation: Supporting; Visualization: Supporting; Writing – review & editing: Supporting
S.F. Formal analysis: Supporting; Investigation: Supporting; Validation: Supporting; Visualization: Supporting; Writing – review & editing: Supporting
J.S. Formal analysis: Supporting; Investigation: Supporting; Methodology: Supporting
G.C. Formal analysis: Supporting; Validation: Supporting; Writing – review & editing: Supporting
Y.Y. Investigation: Supporting; Methodology: Supporting
H.W. Investigation: Supporting; Methodology: Supporting; Writing – review & editing: Supporting
K.T. Investigation: Supporting; Methodology: Supporting
O.L. Funding acquisition: Supporting; Resources: Supporting; Writing – review & editing: Supporting
S.C. Funding acquisition: Supporting; Validation: Supporting
M.H. Funding acquisition: Supporting; Methodology: Supporting; Validation: Equal; Writing – review & editing: Equal
L.L. Conceptualization: Supporting; Data curation: Supporting; Formal analysis: Supporting; Methodology: Supporting; Project administration: Supporting; Validation: Supporting; Writing – original draft: Supporting; Writing – review & editing: Supporting
I.Z. Conceptualization: Supporting; Funding acquisition: Lead; Methodology: Supporting; Project administration: Equal; Resources: Lead; Supervision: Lead; Validation: Supporting; Writing – original draft: Supporting; Writing – review & editing: Equal.

Table of Contents

Material and Methods (pp. S2-S6)

Figure S1 (p. S7)

Figure S2 (p. S8)

Figure S3 (p. S9)

Figure S4 (p.S10)

Figure S5 (p. S11)

Figure S6 (p. S12)

Figure S7 (p. S13)

Figure S8 (p. S14)

Figure S9 (p. S15)

Figure S10 (p. S16)

Figure S11 (p. S17)

Figure S12 (pp. S18-S19)

Figure S13 (p. 20)

Table S1 (p. S21)

Table S2 (p.S21)

References (pp. S22-23)

Material and Methods

Protein purification, crystallization and activity assay

Cell cultivation and protein purification of *Re*RH and *Re*MBH were performed as described by Lenz et al.[1] Protein for NRV spectroscopy was isolated from cells grown in the presence of 18 µM $^{57}FeCl_2$.

Oxidized crystals of *Re*MBH were grown under ambient air from protein purified aerobically as described previously.[1,2] Reduced crystals were grown from the same protein in an anaerobic box (5 % H_2 and 95 % N_2 or 100 % H_2) within 2-3 days.

The specific H_2-oxidizing activity of *Re*RH was quantified photometrically using methylene blue as the electron acceptor, as described by Lenz *et al.*[1] The protein concentration was determined using the Pierce® BCA Protein Assay Kit (Thermo Fisher Scientific, USA).

Lyophilization, concentration and gas exchange

Lyophilization: For *in situ* IR spectroscopy, 3-µl drops of *Re*RH protein solution (0.3 mM) were placed on a 0.5-mm thick CaF_2 window and immediately frozen in liquid N_2. Afterwards, the plate was transferred to the precooled LINKAM cryo stage (80 K) in the sample compartment (**Figure 2A**). After sealing the cryostat air-tight, the pressure was carefully reduced to 0.1 mbar. Subsequently, the temperature was increased by 4 K/min up to 243 K. After 4 hours, the lyophilization process was completed, yielding a sponge-like transparent lyophilizate, which was verified visually *via* the optical microscope. In addition, the sublimation of (IR-absorbing) water molecules was monitored by following the total signal amplitude of a mercury-cadmium-telluride (MCT) detector of a Bruker Tensor 27 FT-IR spectrometer. After a constant signal was reached (indicating completion of the lyophilization procedure), the vacuum valve was closed and the sample chamber purged with dry N_2 gas. The temperature was increase by 20 K/min up to 283 K, and the initial IR spectroscopic measurements of the oxidized sample were performed. Subsequently, the lyophilizate was incubated with H_2 or D_2 while monitoring the redox state composition by IR spectroscopy.

Gas exchange: Before reducing the lyophilized sample by H_2 gas (**Figures 2B**, **3C** and **S3**), the sample compartment was purged with dry N_2 gas for 30 min to remove residual O_2. Afterwards, dried H_2 or D_2 gas streams were passed through the sample compartment to reduce the lyophilizate. After flushing again for 20 minutes with dry N_2 gas, reoxidation was accomplished by purging the chamber with synthetic air (80 % N_2, 20 % O_2). *In situ* redox cycling of the protein solution (**Figures 2B**, **5B**, **S3B**, and **S11B**) and protein crystals (**Figures 2B** and **5A**) was performed in the same manner with humidified gases. For *ex situ* experiments

the oxidized protein solution of *Re*RH (0.3 mM for IR and EPR, 0.5 mM for NRVS) was incubated with humidified H_2 for 1-2 hours for complete reduction (**Figure 3**).

Sample concentration: To obtain highly concentrated protein samples for EPR, RR, and NRV spectroscopic studies, 350 µl (50 µL for RR) of a 0.5 mM *Re*RH solution were filled in a custom-made quartz vessel (**Figure 2C**). Afterwards, samples were lyophilized as described above for 8 h and subsequently reduced for 2-3 h with 100 % dry H_2 or D_2 gas. For further concentration of the lyophilized samples, the portable part of the setup (**Figure S2**) was disconnected and transferred to an anaerobic glovebox. Subsequently, the lyophilizate was compressed mechanically within the sample plate to the maximal possible density using a spatula and mini pestle. For RR spectroscopic analysis, the sample container was frozen in liquid N_2, and for EPR and NRVS characterization the sample was first transferred into a custom-made 30-35 µL NRVS cell sealed with Kapton Tape as described by Wang *et al.*[3] and then frozen in liquid N_2. Since the initial sample volume of 350 µL was reduced by a factor of approximately 10, the initial concentration of 0.5 mM is estimated to be increased up to an equivalent of ca. 4-5 mM.

IR spectroscopy

Transmission cell: *Re*RH protein solution (0.3 mM) was transferred into a homemade, gas-tight IR transmission cell containing two CaF_2 windows separated, typically, by a 50-µm Teflon spacer (**Figure 3C**). For evaluating the integrity of the protein backbone in solution, the optical path length was reduced to 2 µm and the concentration increased to 0.5 mM to monitor amide bands (**Figure 3A**). All IR spectra were recorded with 2 cm^{-1} spectral resolution on a Bruker Tensor 27 FT-IR spectrometer using a liquid-N_2 cooled MCT detector. Absorbance spectra were calculated using the buffer solution (100 mM Tris-HCl, pH 8.0, 150 mM NaCl, 5 mM d-desthiobiotin) as a reference. The Bruker OPUS software 6.5 or higher was used for data evaluation.

IR microscope: To perform *in situ* IR spectroscopy of *Re*MBH and *Re*RH solutions (**Figures 2B**, **5A**, **5B**, **S3**, and **S11**), a 3-µl drop of protein solution (1 mM) was placed on a 1.5 mm thick MgF_2 window. After adding a 40-µM steal spacer, a second 1.5-mm MgF_2 window was carefully placed on top of it in order to form a homogenous protein film. This sandwich cell was then transferred to a precooled liquid-N_2-cooled cryo-stage (Linkam Scientific instruments) at 277-283 K, and IR spectra were recorded *via* a Bruker Tensor 27 FT-IR spectrometer connected to a Bruker Hyperion 3000 IR microscope equipped with a 20× IR transmission objective and an MCT detector. Protein crystals of *Re*MBH (**Figures 2B** and **5A**) were handled the same way, but without using a second window and a steal spacer. For illumination experiments, the focused beam of a collimated 455-nm LED was used. To perform cryogenic IR spectroscopy on H_2-incubated *Re*MBH, protein crystals were mounted on an optical plate in an anaerobic

box and frozen in liquid N_2 until further usage. Protein lyophilizate was prepared as described above, and IR spectra were recorded with a Hyperion 3000 IR microscope. The optical pathlength of the lyophilized drops may vary on a sample-to-sample basis since they shrunk due to water removal to a sponge-like form with undefined dimension.

EPR spectroscopy

A 9.3-GHz X-Band continuous-wave Bruker EMXplus spectrometer equipped with an ER 4122 SHQE resonator was used for recording EPR spectra. The parameters were set as follows: modulation amplitude: 10 G, modulation frequency: 100 kHz, microwave power: 1 mW. An Oxford EPR 900 He-flow cryostat equipped with an Oxford ITC4 controller was used to control the temperature. All data were processed using the Bruker Xenon software (version 1.1b58).

RR Spectroscopy

Resonance Raman spectra were recorded using a LabRam HR-800 Jobin Yvon confocal Raman spectrometer connected to a liquid-N_2-cooled charge-coupled device (CCD). The spectra were accumulated at 80 K using a liquid-N_2-cooled cryo-stage (Linkam Scientific instruments). The 458 and 514-nm lines of an Ar^+ ion laser or the 568-nm line of a Kr^+ ion laser with an overall power of 1-2 mW were used for excitation. The laser beam was focused at a 2-4 µm spot on the surface of the compressed lyophilizate, a frozen drop (3 µL, 1 mM protein concentration) or a single crystal. All RR spectra displayed consist of an average of 30-100 individual spectra, each accumulated for 100-180 s. Frequency calibration was performed using toluene as an external standard.

NRV Spectroscopy

Nuclear resonance vibrational spectroscopy was performed at SPring8 BL19LXU during the bunch mode C (11 bunch train x 29) with a 145.5 ns bunch interval.[4,5] A liquid N_2-cooled Si (1, 1, 1) high heat load monochromator was used to produce an incident beam with ~1 eV resolution, followed by a high resolution monochromator with a Ge (3, 3, 1) and two nested asymmetrically cut Si (9, 7, 5) crystals to achieve a 0.8 meV energy resolution at 14.4125 keV. A 4-channel avalanche photo diode detector array was used to detect the X-ray K-fluorescence from internal conversion and the nuclear gamma ray emission by ^{57}Fe atoms after the beam excitation. The detectors were placed as close as possible to the surface of the sample but outside the cryostat. All measurements were performed at 10 K in the cryostat base. The real sample temperature, as obtained from the spectral analysis, was 30-60 K. To enhance the S/N ratio in the Fe–CO/CN spectral range, sectional measurements of this region were performed. In the case of *Re*RH frozen solution, the acquisition time for each data point was 10 s between 440 and 670 cm^{-1}, 20 s between 670 and 820 cm^{-1}, and 4 s for the remaining part of the

spectrum. In case of the lyophilized *Re*RH, the acquisition time was 6 s between 400 and 640 cm^{-1}, 24 s between 640 and 830 cm^{-1}, and 1 s for the remaining part of the spectrum. In total, 12 scans were measured for *Re*RH frozen solution, 26 scans for the H_2-reduced and lyophilized sample and 16 scans for the D_2-reduced and lyophilized sample. The raw NRVS data were analyzed by the PHOENIX software and "spectra.tools" [6,7] to extract the single-phonon ^{57}Fe partial vibrational density of states (^{57}Fe-PVDOS). The resonance peak position for each scan was aligned in the PHOENIX analysis, while the energy scale was calibrated with an external reference (signal from $[NEt_4][FeCl_4]$ at 380 cm^{-1}). The S/N ratios of lyophilized and frozen-solution *Re*RH were compared at an energy range with similar accumulation times (670 to 750 cm^{-1}). The lyophilizate did not form a homogeneous phase but a sponge-like phase containing air pockets in the NRVS cell, while the frozen-solution sample was homogeneous and well packed. Accordingly, the count rate of the NRVS signal for the lyophilized sample may have underestimated the actual sample concentration, as the synchrotron beam might also have probed empty space.

DFT Calculations

In lack of an experimentally resolved *Re*RH protein structure, initial coordinates used for DFT calculations were based on crystallographic X-ray data of H_2-reduced *Re*MBH (PDB 3RGW, 1.50 Å resolution).[8] Among the [NiFe]-hydrogenases with their X-ray structures available, the residues forming the [NiFe] cofactor pocket in *Re*MBH (HoxG protein) were found to provide the closest (aligned) sequence match to *Re*RH (HoxC protein). The DFT model (**Figure S5**) included the [NiFe] core together with its metal ligands and all side chains exhibiting non-hydrogen interatomic distances within 4 Å from the first-shell Ni/Fe ligand nuclei. At this modeling level, three amino acid variations were identified between the large subunits of *Re*MBH (HoxG)[9] and *Re*RH (HoxC).[10] Based on the PDB 3RGW coordinates, the model was prepared by means of virtual HoxG-to-HoxC modifications: His82-to-Gln67, Pro529-to-Ala410, and Val551-to-Ala432. All C_α carbon nuclei appearing in the above-described *Re*RH homology model were locked to their original X-ray positions during structure optimizations. These optimizations were accomplished using GAUSSIAN 09 Revision D.01[11] based on high-quality initial guess from single-point calculations using JAGUAR 9.4.[12] The calculations employed the BP86 functional[13,14] in its unrestricted formulation, and the LACV3P** basis set as implemented in JAGUAR 9.4. For the first- and second-row elements, LACV3P** implies the 6-311G** triple-ζ basis sets including polarization functions. For the Fe atoms, LACV3P** consists of a triple-ζ basis set for the outermost core and valence orbitals, and the quasi-relativistic Los Alamos effective core potential (ECP) for the innermost electrons.[15,16] The two-body D3 dispersion corrections by Grimme et al.[17,18] as implemented in GAUSSIAN 09 were included. The molecular system's environment was considered using a self-consistent reaction

field (SCRF) polarizable continuum model and integral equation formalism (IEF-PCM)[19] with the static dielectric constant set to ε = 4.0 as often used for proteins and the remaining IEF-PCM parameters at their default values for water. The ^{57}Fe-PVDOS (simulated NRVS) intensities were extracted from GAUSSIAN 09 normal mode outputs using an in-house program Q-SPECTOR, successfully applied in our previous works. To empirically account for the observed NRVS lineshape, the computed ^{57}Fe-PVDOS intensities were broadened by Lorentzian convolution with a full width at half maximum (FWHM) = 12 cm^{-1}. An empirical scaling by ×0.95 and ×0.98 was applied to the calculated frequencies in the 400-500 cm^{-1} and > 500 cm^{-1} regions, respectively, relying on one-to-one mapping between the observed and simulated bands.

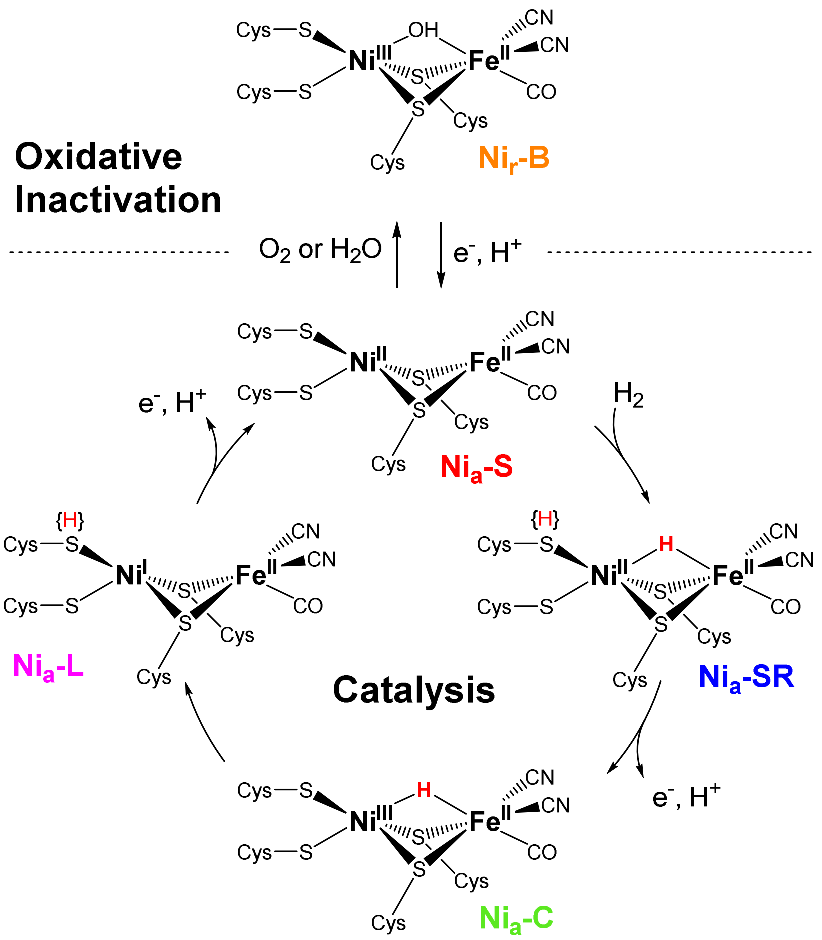

Figure S1: Proposed catalytic cycle of H_2 conversion and oxidative inactivation of O_2-tolerant [NiFe]-hydrogenases. The colored names of the different redox states are used for state assignment in the displayed IR, EPR and RR spectra. The subscribed indices “a” for active and “r” for ready denote catalytic active states and inhibited resting states that can be quickly reactivated, respectively. Additionally, the $Ni_{u/r}$-S (u/r = unready/ready) and Ni_{ia}-S (ia = inactive) states can be identified *via* their characteristic CO and CN stretching frequencies.[20,21] Due to the unknown structure and function of these states, they are not displayed here. Within the proposed catalytic cycle, H_2 binds to the Ni_a-S state, containing a vacant coordination site between the two metal ions. Splitting of H_2 could be accomplished either by heterolytic cleavage or oxidative addition.[22] Thereby, Ni_a-SR, with a Ni^{2+} ion, a bridging hydride between the two metal ions, and presumably a proton on the terminal cysteine, is formed.[23] Subsequently, one electron is released to the electron transfer chain, and a proton is presumably transferred to a conserved adjacent glutamate residue thereby forming the Ni_a-C state, with a Ni^{3+} ion, *vide supra*.[24] *Via* tautomerization, Ni_a-C is transformed to the related Ni_a-L state, harboring a formal Ni^{1+} ion and a protonated terminal cysteine.[25,26] Finally, this species is converted by a concerted $1H^+/1e^-$ step to the Ni_a-S state, closing the catalytic cycle and providing access for the cleavage of a further hydrogen molecule.[24,27] Upon oxidative inactivation (e.g. under aerobic conditions) the active site converts to the Ni_r-B state containing a bridging hydroxide species. Additionally, several so called Ni_r-S species, featuring supposedly an oxygen-derived ligand at or between Ni^{2+} and Fe^{2+} ion, have been characterized under aerobic conditions. Their exact structure and function are still under debate.[28–31]

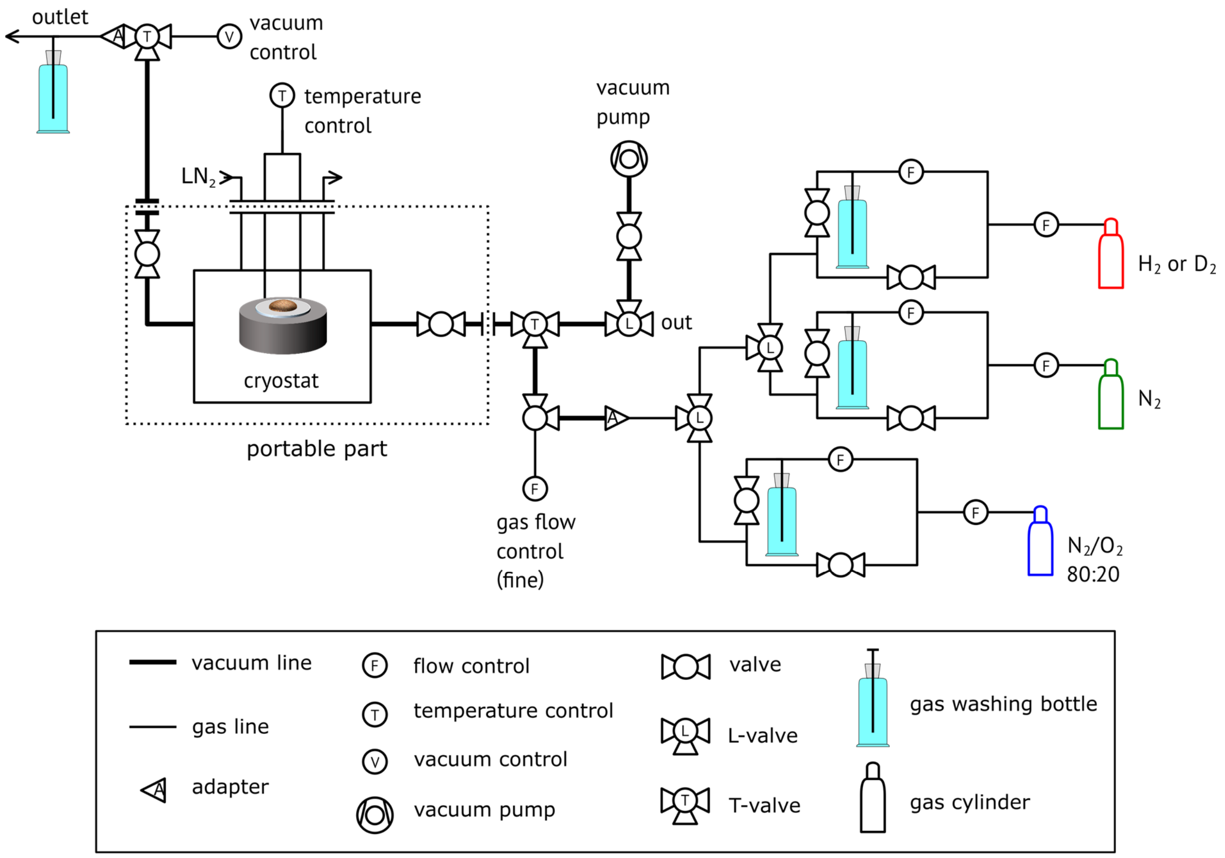

Figure S2: Flow chart of the new experimental setup used for *in situ* IR spectroscopy.

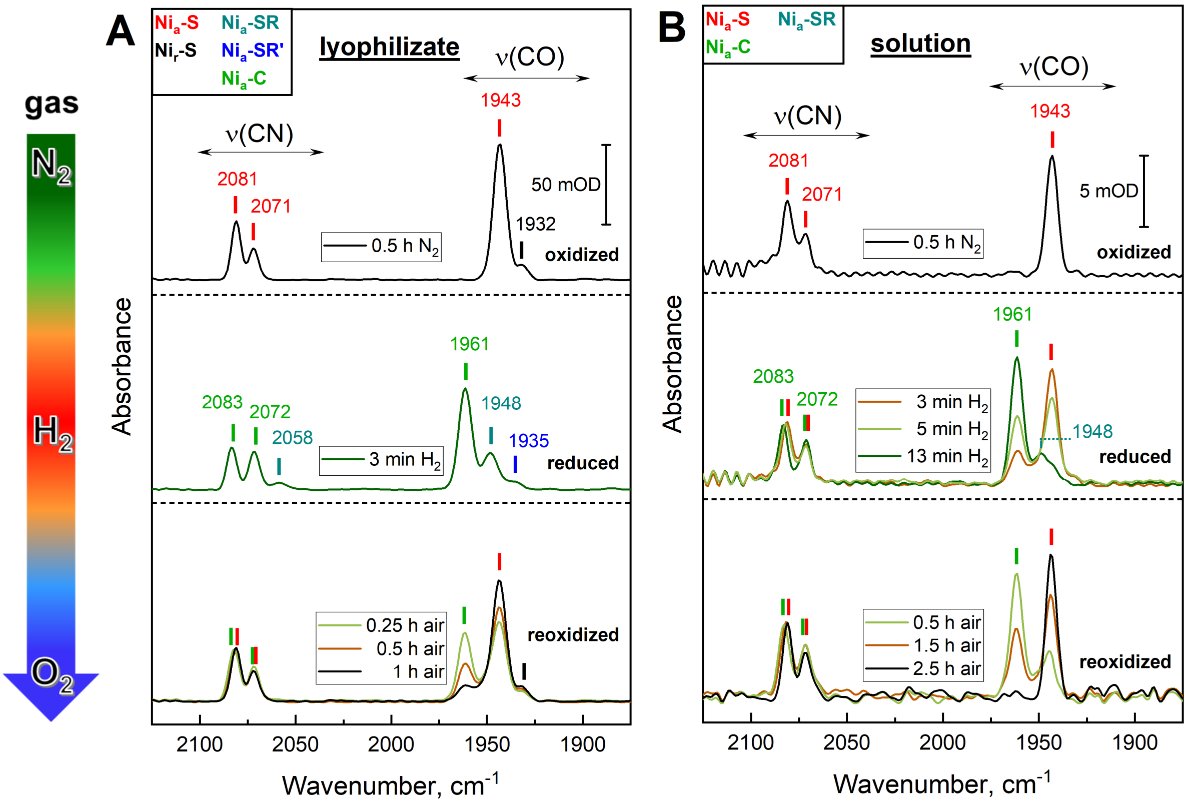

Figure S3: *In situ* IR spectroscopy of *Re*RH lyophilizate and solution. IR spectra of oxidized (top), reduced (middle) and re-oxidized (bottom) enzyme after consecutive exposure to various humidified gases (N_2, H_2, synthetic air) at 283 K. **(A)** Lyophilized *Re*RH mounted on a single MgF_2 plate (**Figure 2B**) instantly reacts with H_2, resulting in complete reduction after 3 minutes. In contrast, re-oxidation by synthetic air takes up to one hour. The slower diffusion of O_2 through the narrowed gas channel of *Re*RH – supposedly responsible for its O_2 tolerance – can explain this observation. **(B)** *Re*RH solution displays similar relative redox kinetics. The design of the sandwich cell (**Figure 2B**) allows only slow gas diffusion to the protein film and is probably the reason for the 3-4 times slower reaction of *Re*RH solution with H_2 or O_2.

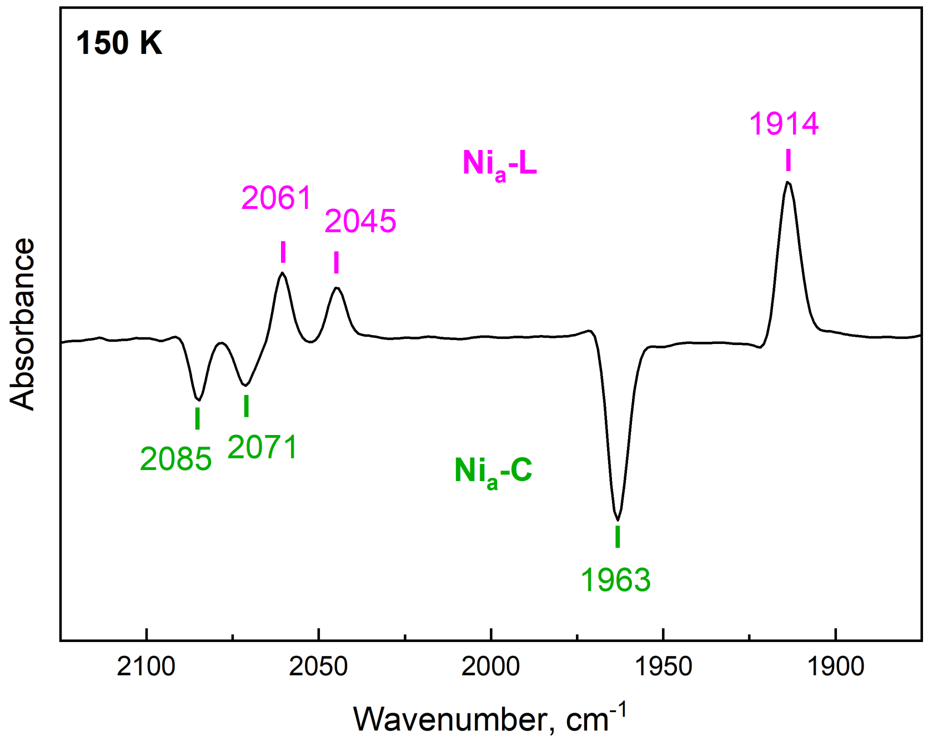

Figure S4: Photoreaction of the Ni$_a$-C state of *Re*RH. Light-*minus*-dark IR difference spectrum recorded at 150 K in the sandwich-cell configuration of the setup (Figure 2B). After enriching the Ni$_a$-C state by H_2 reduction, the sample was frozen inside the sample compartment. Upon illumination with a 455-nm LED, the formation of a homogenous Ni$_a$-L state was observed.

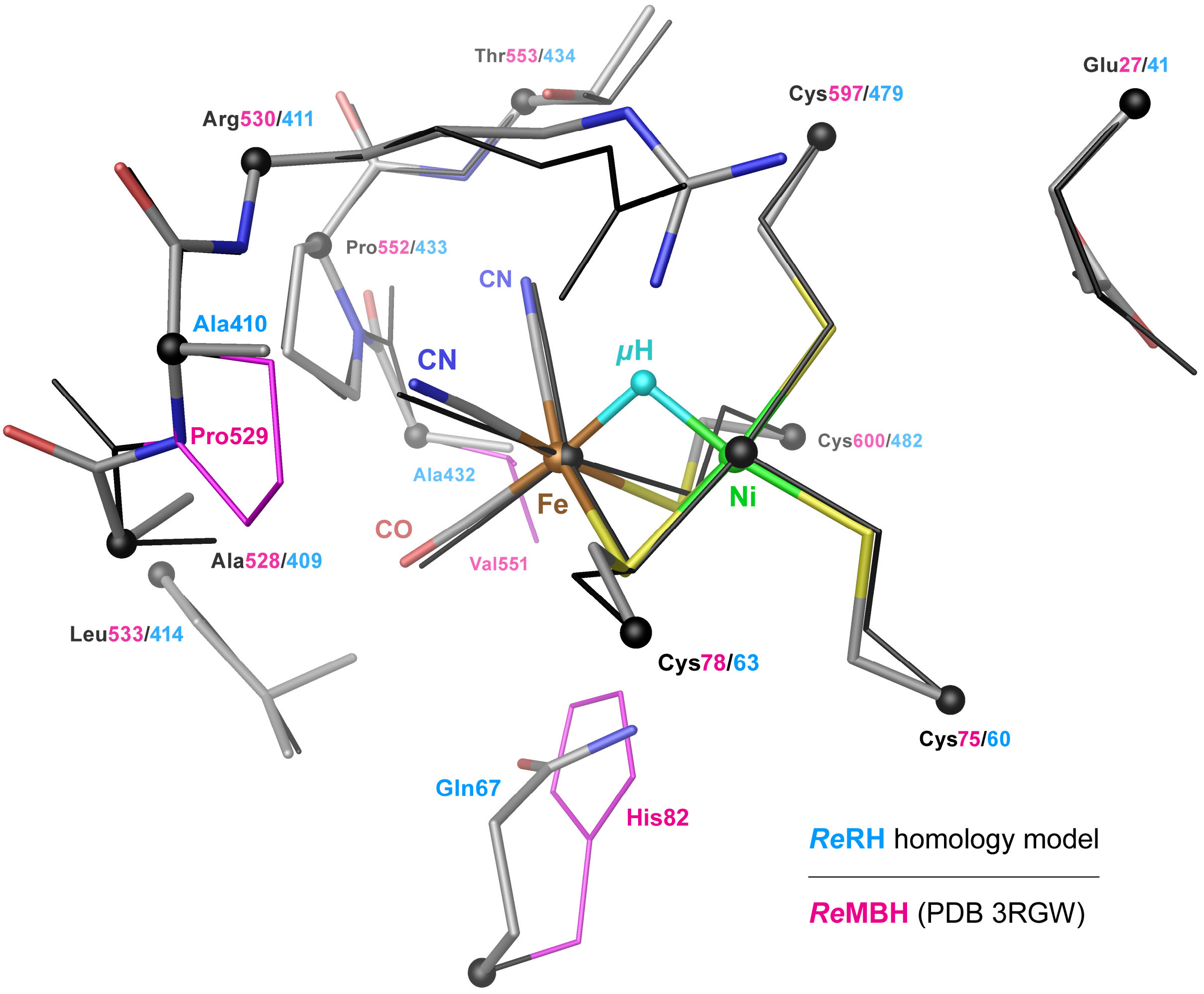

Figure S5: Homology model of the *Re*RH active site. The DFT model optimized in the Ni_a-C state (element colors, thick tubes) was superimposed onto its reference X-ray structure of the H_2-reduced *Re*MBH (PDB 3RGW,[8] black thin tubes). The side chain labels shown correspond to their protein sequence numbers in the large subunits of *Re*MBH (HoxG) / *Re*RH (HoxC) (magenta/blue). The *Re*MBH-to-*Re*RH amino acid modifications are highlighted in magenta tubes for *Re*MBH: His82-to-Gln67, Pro529-to-Ala410, and Val551-to-Ala432. C_α carbon nuclei (black), the two metal centers, and the bridging μH^- (μD^-) ligand are shown in ball representation. The other hydrogen atoms are omitted for clarity; see **Figure S6** for the DFT Ni_a-C structure including protons.

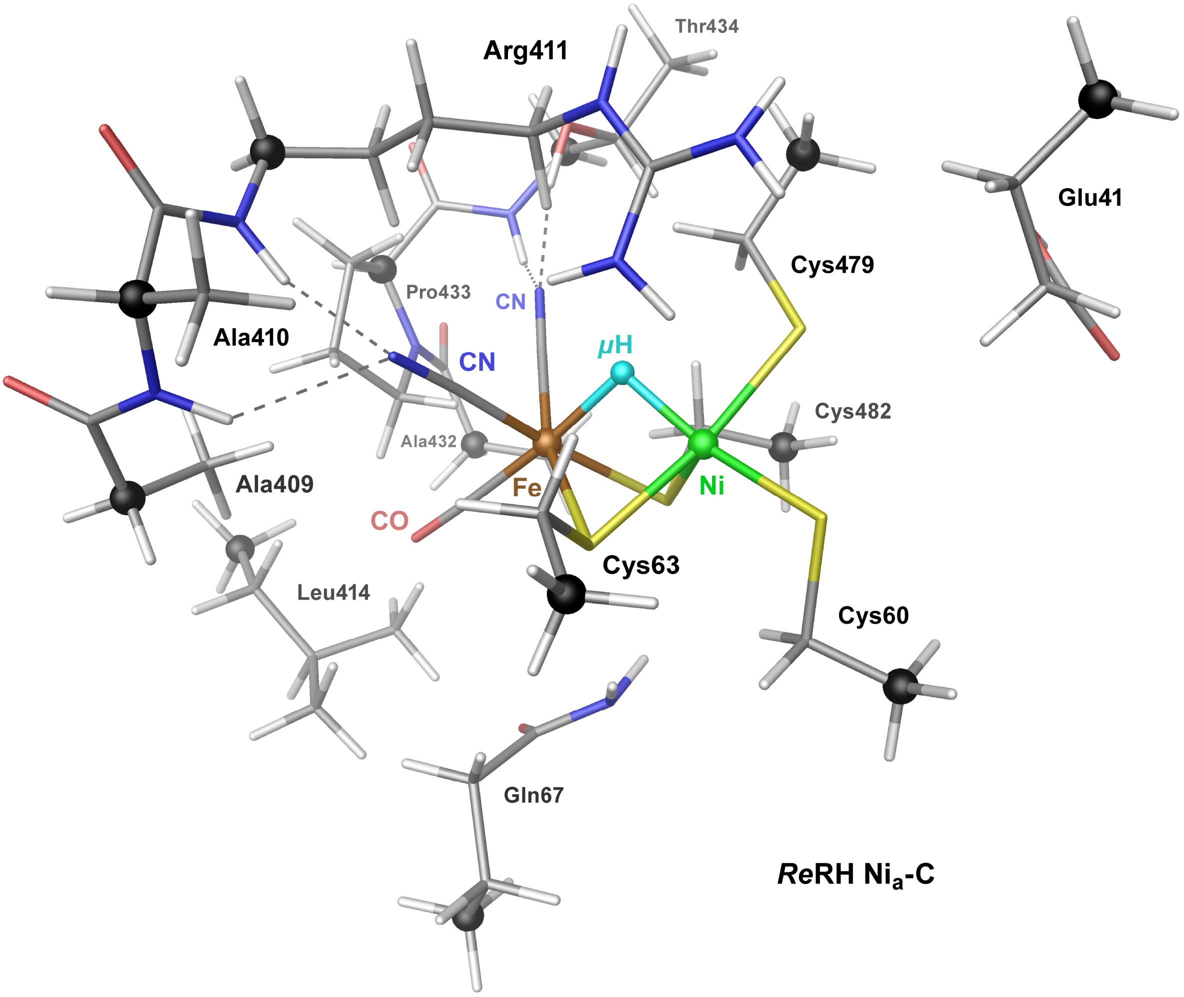

Figure S6: DFT-optimized model of the *Re*RH active site in the Ni$_a$-C state containing a bridging hydride ligand. C$_\alpha$ carbon nuclei (black, kept fixed during the structure optimizations), the two metal centers, and the bridging μH$^-$ (μD$^-$) ligand are shown in ball representation. Cartesian coordinates for the model shown are available as a separate part of the Supporting Information.

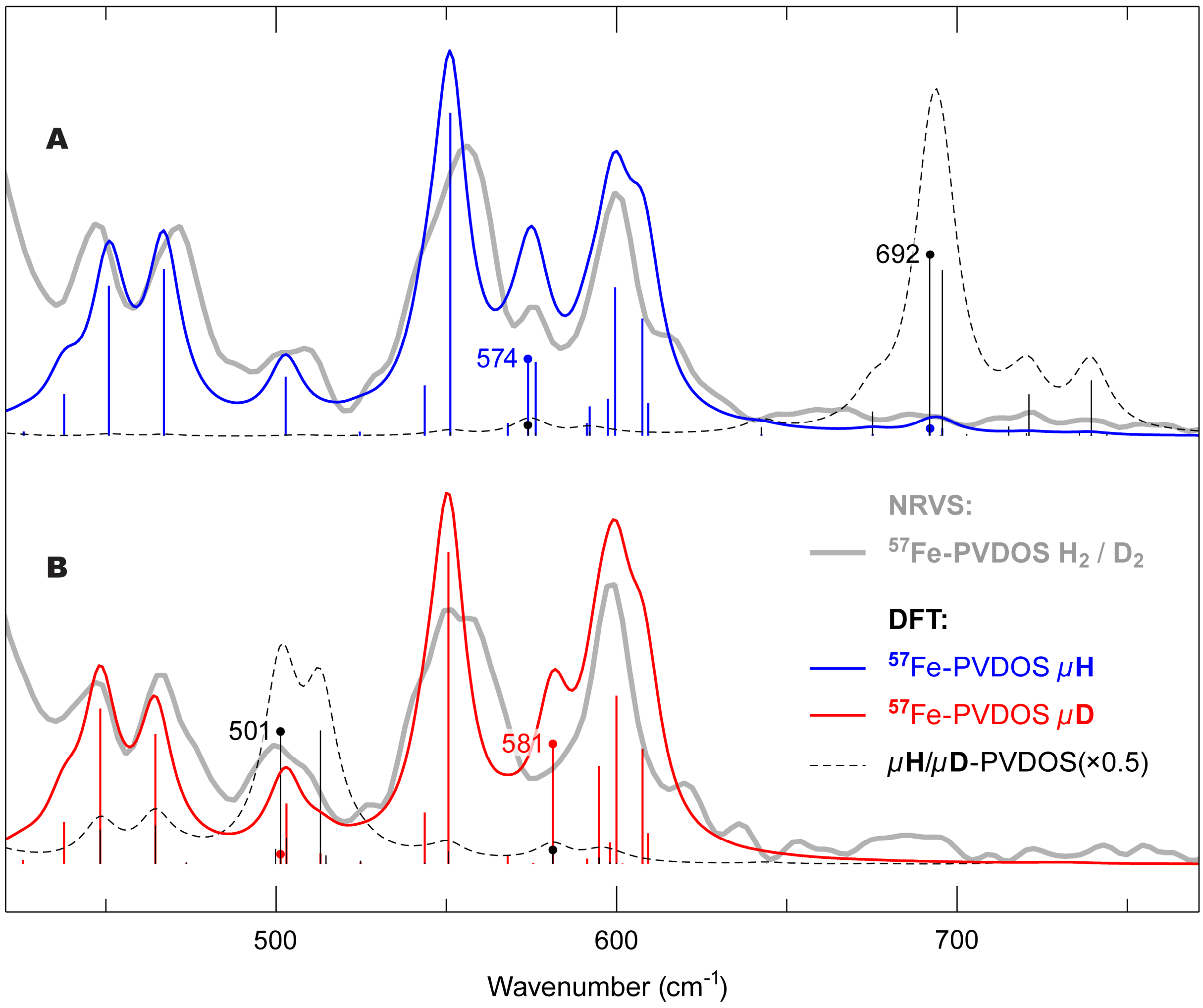

Figure S7: ^{57}Fe- and *μ*H/*μ*D-PVDOS spectra of *Re*RH in the Ni$_a$-C state. The DFT-predicted ^{57}Fe-PVDOS spectra for both (**A**) hydride *μ*H and (**B**) deuteride *μ*D metal-bridging ligand isotopologues of Ni$_a$-C are overlaid with the NRVS-observed data from the (**A**) H_2- and (**B**) D_2-reduced enzyme samples, respectively. The bridging ligand nucleus *μ*H/*μ*D-PVDOS, available from computations only, is shown scaled to 50 % relative to the iron nucleus ^{57}Fe-PVDOS. Stick-style DFT PVDOS data are provided additionally to the homogeneously broadened (FWHM = 12 cm^{-1}) spectra, displaying individual normal mode frequencies and their relative intensities. The sticks labeled with dots and vibrational energies (cm^{-1}) correspond to four modes displayed in **Figure S8**.

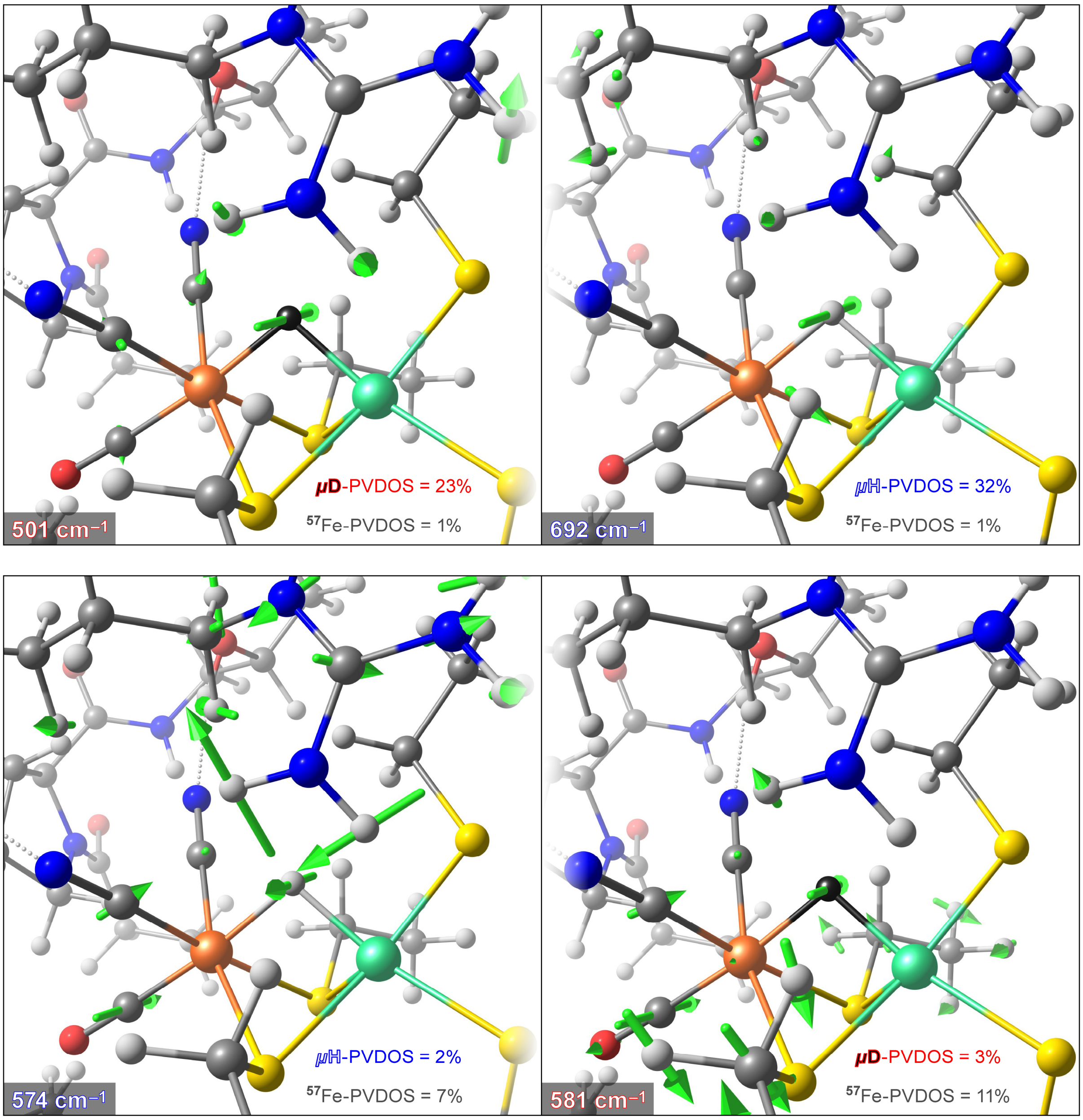

Figure S8: Arrow-style representation of selected normal modes of the *Re*RH Ni$_a$-C state from DFT modeling. Top: the two normal modes with strongest Ni-μD-Fe (left) and Ni-μH-Fe (right) wagging motion character. Bottom: the two normal modes underlying the difference between the ^{57}Fe-PVDOS (NRVS) spectra of the μH/μD Ni$_a$-C isotopologues in the 570-590 cm^{-1} region (as shown in **Figure S7** and **Figure 4B**). The deuteride μD bridging ligand is shown in black. Mode-specific PVDOS contributions from the μH/μD and ^{57}Fe nuclei are provided (%). Animated representations of these and other μH/μD Ni$_a$-C normal modes relevant to the ^{57}Fe-PVDOS analysis (440-740 cm^{-1} range, **Figure S7**) are available as part of the Supporting Information separately.

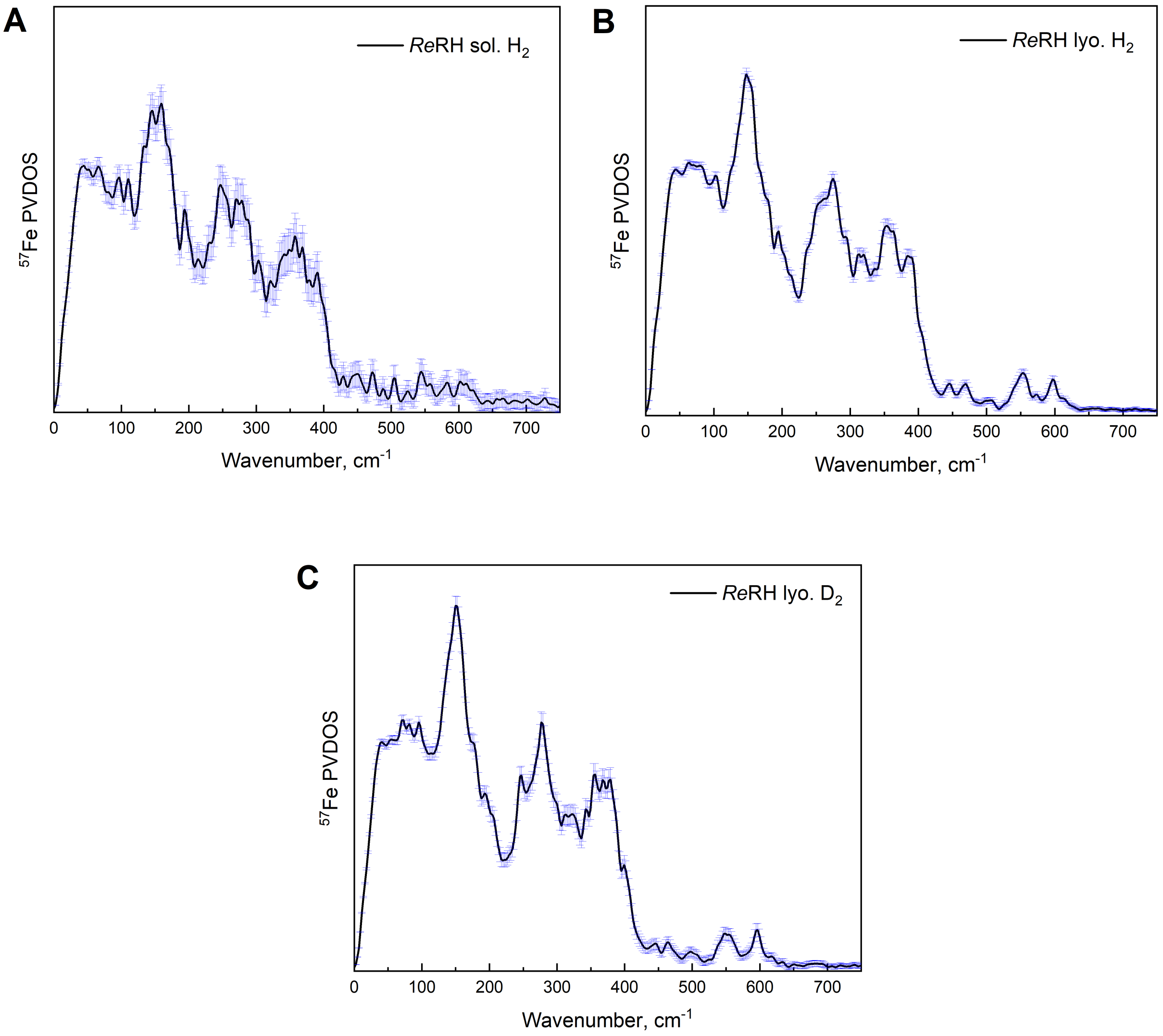

Figure S9: Nuclear resonance vibrational spectra of *Re*RH including error bars. (**A**) NRVS data of solvated (sol.) and H_2-reduced *Re*RH, (**B**) lyophilized (lyo.), H_2-reduced and compressed *Re*RH and (**C**) lyophilized, D_2-reduced and compressed *Re*RH after 12, 26 and 16 hours of accumulation, respectively.

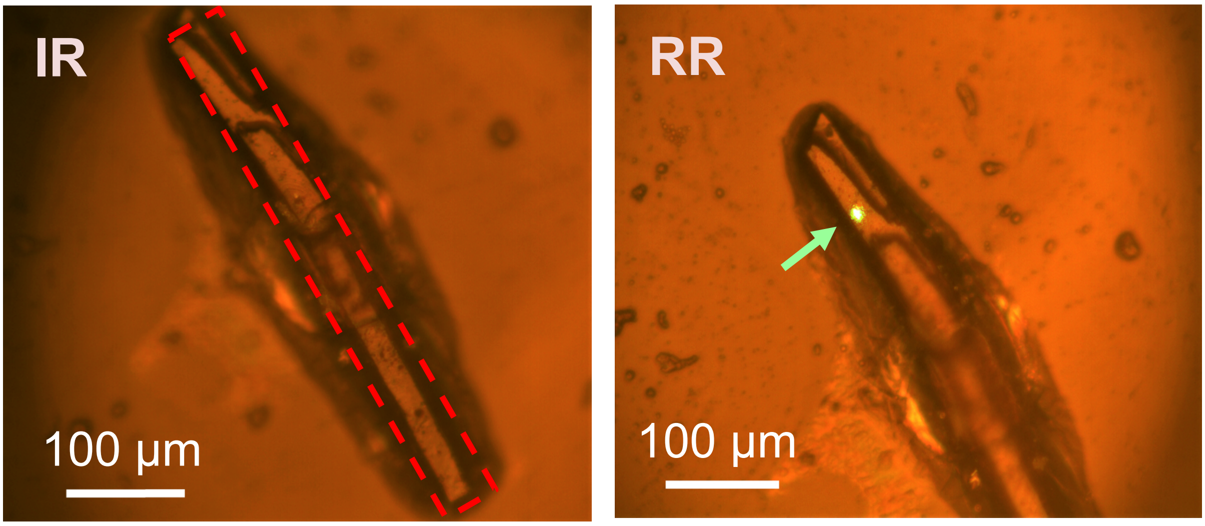

Figure S10: Optical microscope photographs showing a *Re*MBH crystal and the areas probed by IR and RR spectroscopy. The red frame (left figure) indicates the probed area for the IR transmission experiment. Two pairs of movable non-IR-transparent filters can be adjusted to select the correct window (red frame) depending on crystal size and shape. The small 514-nm laser spot used for the RR experiment (right figure) is indicated by a green arrow.

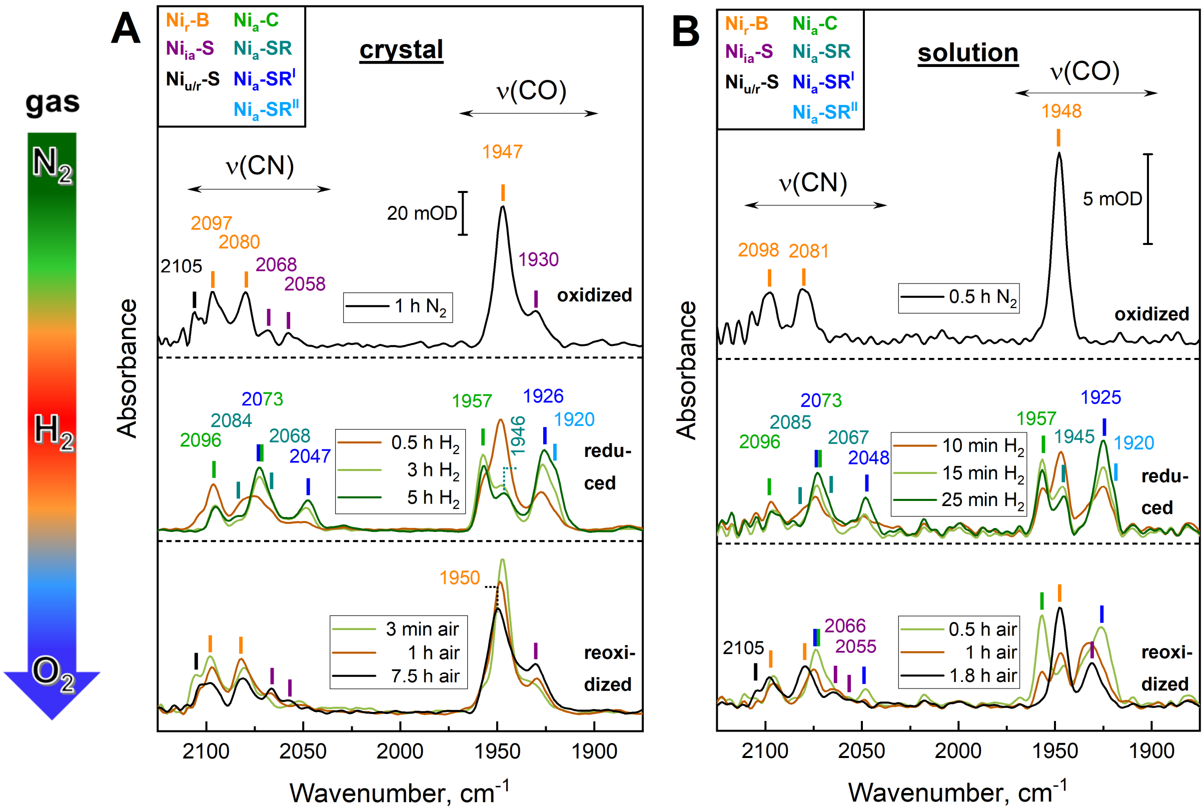

Figure S11: *In situ* IR spectroscopy of *Re*MBH crystals and solution. IR spectra of oxidized (top), reduced (middle), and re-oxidized (bottom) enzyme crystals (**A**) and solution (**B**) after consecutive exposure to various humidified gases (N_2, H_2, synthetic air) at 277 K. As described in the caption of **Figure S3**, the design of the sandwich cell (**Figure 2B**) might slow down gas diffusion for the experiments in solution compared to the protein crystal exposed on a single optical plate (**Figure 2B**). Exposing *Re*MBH crystals to air over a long time (**A**, lower traces) led to the gradual decrease and blue shift of the CO band at 1947 cm^{-1}, assigned to the Ni_r-B state, and a concomitant increase of a signal at 1930 cm^{-1}, related to an inactive Ni_{ia}-S state.

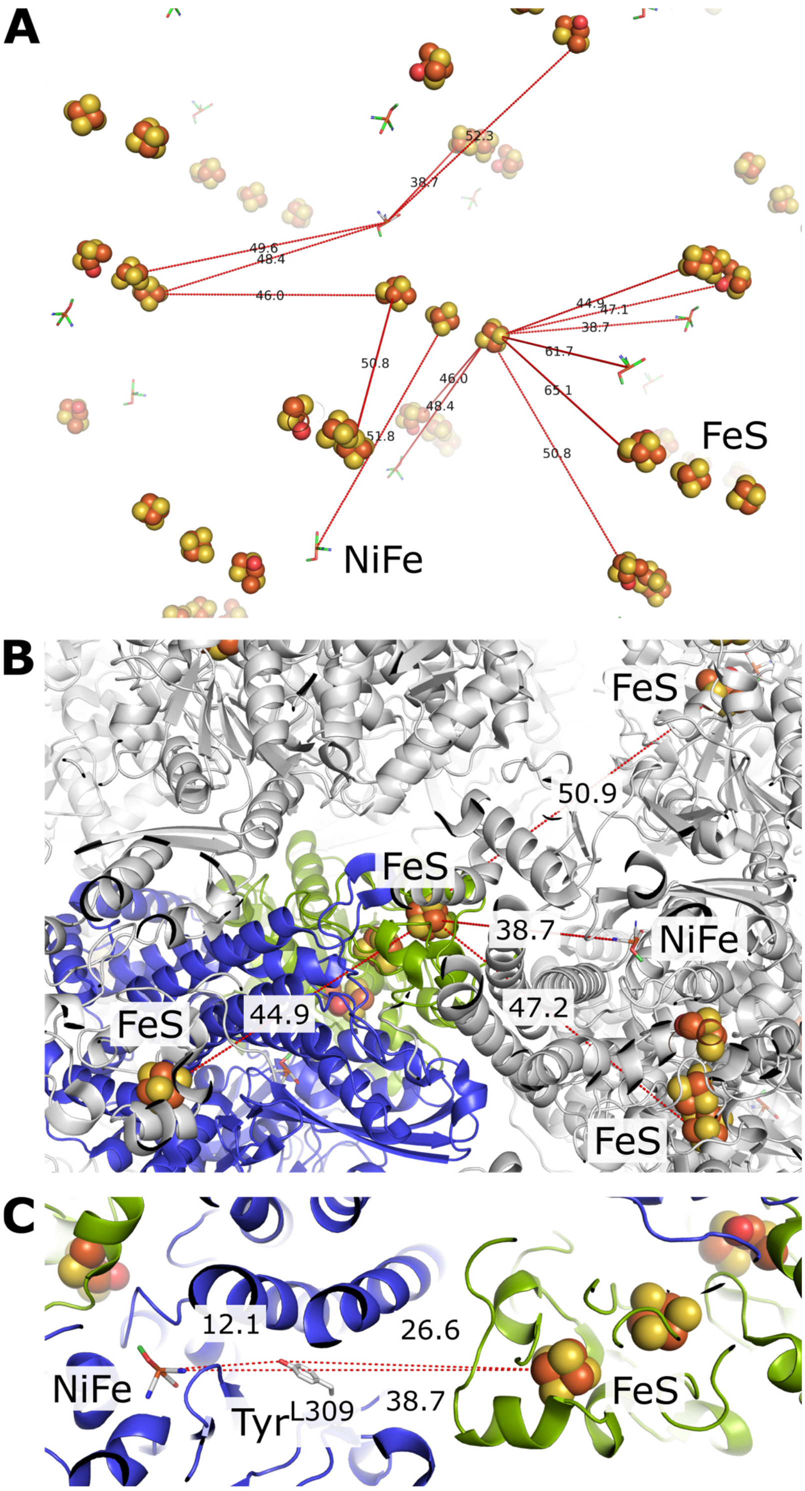

Figure S12: Distances for putative long-range intermolecular electron transfer steps in *Re*MBH crystals. Sulfur atoms are displayed as yellow, iron as orange, proximal cluster coordinating oxygen as red spheres. The active site is depicted as sticks. (**A**) Distances between metal clusters of different *Re*MBH molecules. (**B**) Shortest distances of the distal cluster to close-by metal clusters. (**C**) Shortest distance between the distal cluster and the active site of another *Re*MBH molecule exhibiting an interjacent tyrosine residue.

In *Re*MBH crystals, each protein heterodimer is locked in its position, and its orientation towards neighboring molecules is fixed. Therefore, random interactions between different MBH molecules – as found in solution – can be excluded. However, we still observed slow H_2-mediated MBH activation starting from the Ni_r-B state. Either, Ni_r-B is activated directly by H_2[33] or a minor fraction of MBH molecules in the Ni_a-S state activate neighboring MBH molecules. The latter scenario depends on electron transfer events that typically occur fast in the range of (10-15 Å).[34] In order to evaluate the feasibility of intermolecular electron transfer events in MBH crystals, MBH symmetry mates (PDB code 4IUC)[2] were generated using PyMOL.[35] The shortest distance between two metal clusters was 38.7 Å between the distal FeS cluster of one MBH and the [NiFe] site of a neighboring MBH molecule. However, tyrosine and other residues may enhance electron transfer over longer distances.[36,37] Intriguingly, a tyrosine residue occurs in the intercept between the active site and the distal cluster of a neighboring *Re*MBH unit. The presence of such an amino acid raises the question whether tyrosine-mediated electron/hole-hopping may enable Ni_r-B activation in *Re*MBH crystals.[36,38]

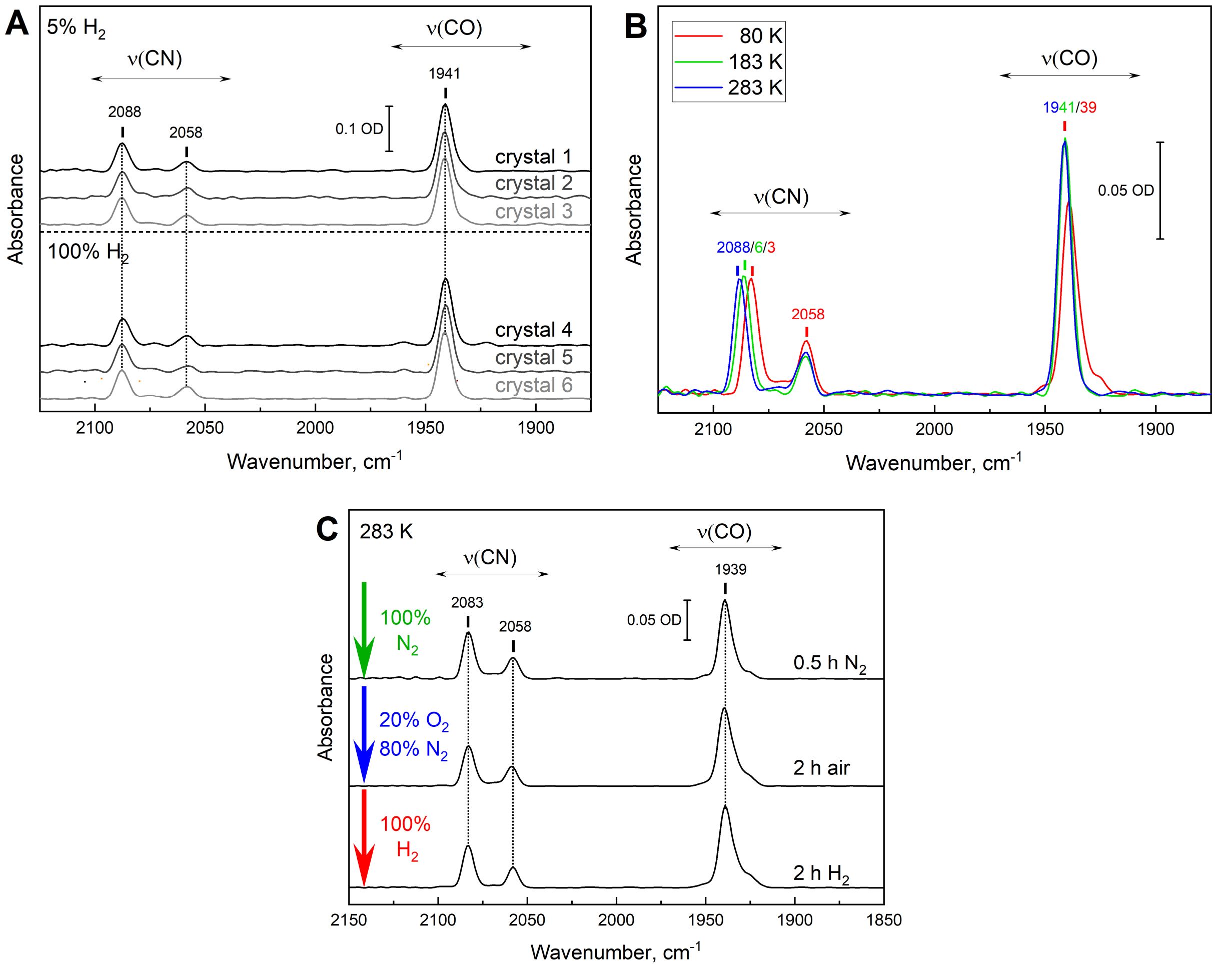

Figure S13: **IR spectra of H_2-reduced *Re*MBH crystals.** (**A**) IR spectra from crystals grown under 5 % H_2 and 95 % N_2 (top traces) and 100 % H_2 (bottom traces) recorded at 80 K. (**B**) Temperature-dependent IR spectra of one crystal grown under 5 % H_2 and 95 % N_2. (**C**) IR spectra of the same crystal consecutively incubated with N_2 (0.5 h), synthetic air (2 h), and H_2 (2 h).

Table S1: CO and CN stretching frequencies of *Re*RH redox states observed in this study.

State	ν(CO) / cm^{-1}	ν(CN) / cm^{-1}		Reference
Ni_r-S	1932	n.d.	n.d.	[39]
Ni_a-S	1943	2072	2082	[32,39,40]
Ni_a-C	1961	2072	2083	[32,39,40]
Ni_a-L	1914*	2045*	2061*	This work
Ni_a-SR	1948	2058	n.d.	[32,39]
Ni_a-SR’	1935	n.d.	n.d.	[39]

n.d. not detected

* Band positions obtained from cryogenic IR (150 K)

Table S2: CO and CN stretching frequencies of *Re*MBH redox states observed in this study.

State	ν(CO) / cm^{-1}	ν(CN) / cm^{-1}		Reference
Ni_r-B	1948	2081	2098	[21,28,41]
$Ni_{u/r}$-S	1943	2082	2105	[21,28,41]
Ni_{ia}-S	1930	2058	2068	[21,28,41]
Ni_a-C	1957	2073	2096	[21,28,41]
Ni_a-SR	1946	2068	2085	[21,28,41]
N_{ia}-SR’	1925	2048	2073	[21,28,41]
Ni_a-SR’’	1920	2048	2073	[21,28,41]
unknown	1939	2058	2083	This work
unknown (light-induced)	1924*<br>1935*	2067*	2078*	This work

* Band positions obtained from cryogenic IR (80 K)

References

[1] O. Lenz, L. Lauterbach, S. Frielingsdorf, *Methods Enzymol.* **2018**, *613*, 117–151.

[2] S. Frielingsdorf, J. Fritsch, A. Schmidt, M. Hammer, J. Löwenstein, E. Siebert, V. Pelmenschikov, T. Jaenicke, J. Kalms, Y. Rippers, F. Lendzian, I. Zebger, C. Teutloff, M. Kaupp, R. Bittl, P. Hildebrandt, B. Friedrich, O. Lenz, P. Scheerer, *Nat. Chem. Biol.* **2014**, *10*, 378–85.

[3] H. Wang, E. Alp, Y. Yoda, S. P. Cramer, *Methods Mol. Biol.* **2014**, *1122*, 125–137.

[4] L. Lauterbach, H. Wang, M. Horch, L. B. Gee, Y. Yoda, Y. Tanaka, I. Zebger, O. Lenz, S. P. Cramer, *Chem. Sci.* **2015**, *6*, 1055–1060.

[5] L. Lauterbach, L. B. Gee, V. Pelmenschikov, F. E. Jenney, S. Kamali, Y. Yoda, M. W. W. Adams, S. P. Cramer, *Dalt. Trans.* **2016**, *45*, 7215–7219.

[6] W. Sturhahn, *Hyperfine Interact.* **2000**, *125*, 149–172.

[7] L. B. Gee, H. Wang, S. P. Cramer, *Methods Enzymol.* **2018**, *599*, 409–425.

[8] J. Fritsch, P. Scheerer, S. Frielingsdorf, S. Kroschinsky, B. Friedrich, O. Lenz, C. M. T. Spahn, *Nature* **2011**, *479*, 249–252.

[9] C. Kortluke, K. Horstmann, E. Schwartz, M. Rohde, R. Binsack, B. Friedrich, *J. Bacteriol.* **1992**, *174*, 6277–6289.

[10] E. Schwartz, A. Henne, R. Cramm, T. Eitinger, B. Friedrich, G. Gottschalk, *J. Mol. Biol.* **2003**, *332*, 369–383.

[11] M. J. Frisch, G. W. Trucks, H. B. Schlegel, G. E. Scuseria, M. A. Robb, J. R. Cheeseman, G. Scalmani, V. Barone, B. Mennucci, G. A. Petersson, H. Nakatsuji, M. Caricato, X. Li, H. P. Hratchian, A. F. Izmaylov, J. Bloino, G. Zheng, J. L. Sonnenberg, M. Hada, M. Ehara, K. Toyota, R. Fukuda, J. Hasegawa, M. Ishida, T. Nakajima, Y. Honda, O. Kitao, H. Nakai, T. Vreven, J. A. Montgomery, Jr., J. E. Peralta, F. Ogliaro, M. Bearpark, J. J. Heyd, E. Brothers, K. N. Kudin, V. N. Staroverov, R. Kobayashi, J. Normand, K. Raghavachari, A. Rendell, J. C. Burant, S. S. Iyengar, J. Tomasi, M. Cossi, N. Rega, J. M. Millam, M. Klene, J. E. Knox, J. B. Cross, V. Bakken, C. Adamo, J. Jaramillo, R. Gomperts, R. E. Stratmann, O. Yazyev, A. J. Austin, R. Cammi, C. Pomelli, J. W. Ochterski, R. L. Martin, K. Morokuma, V. G. Zakrzewski, G. A. Voth, P. Salvador, J. J. Dannenberg, S. Dapprich, A. D. Daniels, Ö. Farkas, J. B. Foresman, J. V. Ortiz, J. Cioslowski and D. J. Fox,, *Gaussian 09, Revision D.01, Gaussian Inc., Wallingford CT,* **2009**.

[12] *Jaguar version 9.4, Schrodinger, Inc., New York, NY* **2016**.

[13] A. D. Becke, *Phys. Rev A* **1988**, *38*, 3098–3100.

[14] J. P. Perdew, *Phys. Rev. B* **1986**, *33*, 8822–8824.

[15] R. Krishnan, J. S. Binkley, R. Seeger, J. A. Pople, *J. Chem. Phys.* **1980**, *72*, 650–654.

[16] A. D. McLean, G. S. Chandler, *J. Chem. Phys.* **1980**, *72*, 5639–5648.

[17] S. Grimme, J. Antony, S. Ehrlich, H. Krieg, *J. Chem. Phys.* **2010**, *132*.

[18] L. Goerigk, S. Grimme, *Phys. Chem. Chem. Phys.* **2011**, *13*, 6670–6688.

[19] J. Tomasi, B. Mennucci, R. Cammi, *Chem. Rev.* **2005**, *105*, 2999–3093.

[20] M. Saggu, I. Zebger, M. Ludwig, O. Lenz, B. Friedrich, P. Hildebrandt, F. Lendzian, *J. Biol. Chem.* **2009**, *284*, 16264–16276.

[21] M. Saggu, M. Ludwig, B. Friedrich, P. Hildebrandt, R. Bittl, F. Lendzian, O. Lenz, I. Zebger, *ChemPhysChem* **2010**, *11*, 1215–1224.

[22] M. Bruschi, M. Tiberti, A. Guerra, L. De Gioia, *J. Am. Chem. Soc.* **2014**, *136*, 1803–1814.

[23] H. Ogata, K. Nishikawa, W. Lubitz, *Nature* **2015**, *520*, 571–4.

[24] H. Tai, K. Nishikawa, Y. Higuchi, Z. wan Mao, S. Hirota, *Angew. Chemie - Int. Ed.* **2019**, *58*, 13285–13290.

[25] H. Tai, K. Nishikawa, M. Suzuki, Y. Higuchi, S. Hirota, *Angew. Chemie - Int. Ed.* **2014**, *53*, 13817–13820.

[26] R. Hidalgo, P. A. Ash, A. J. Healy, K. A. Vincent, *Angew. Chemie - Int. Ed.* **2015**, *127*, 7216–7219.

[27] B. L. Greene, G. E. Vansuch, B. C. Chica, M. W. W. Adams, R. B. Dyer, *Acc. Chem. Res.* **2017**, 2718–2726.

[28] M. Saggu, I. Zebger, M. Ludwig, O. Lenz, B. Friedrich, P. Hildebrandt, F. Lendzian, *J. Biol. Chem.* **2009**, *284*, 16264–16276.

[29] W. Lubitz, H. Ogata, O. Rüdiger, E. Reijerse, *Chem. Rev.* **2014**, *114*, 4081–4148.

[30] G. Caserta, C. Lorent, A. Ciaccafava, M. Keck, R. Breglia, C. Greco, C. Limberg, P. Hildebrandt, S. P. Cramer, I. Zebger, O. Lenz, *Chem. Sci.* **2020**, *11*, 5453–5465.

[31] G. Caserta, V. Pelmenschikov, C. Lorent, A. F. T. Waffo, S. Katz, L. Lauterbach, J. Schoknecht, H. Wang, Y. Yoda, K. Tamasaku, M. Kaupp, P. Hildebrandt, O. Lenz, S. P. Cramer, I. Zebger, *Chem. Sci.* **2021**

[32] T. Buhrke, O. Lenz, N. Krauss, B. Friedrich, *J. Biol. Chem.* **2005**, *280*, 23791–23796.

[33] S. Kurkin, S. J. George, R. N. F. Thorneley, S. P. J. Albracht, *Biochemistry* **2004**, *43*, 6820–6831.

[34] H. B. Gray, J. R. Winkler, *Annu. Rev. Biochem.* **1996**, *65*, 537–561.

[35] PyMOL Molecular Graphics System, *Version 2.3.3* **2019**, *LLC.*, Schrodinger.

[36] H. B. Gray, J. R. Winkler, *Proc. Natl. Acad. Sci.* **2005**, *102*, 3534–3539.

[37] H. B. Gray, J. R. Winkler, *Proc. Natl. Acad. Sci.* **2015**, *112*, 10920–10925.

[38] F. A. Tezcan, B. R. Crane, J. R. Winkler, H. B. Gray, *Proc. Natl. Acad. Sci.* **2001**, *98*, 5002–5006.

[39] P. A. Ash, J. Liu, N. Coutard, N. Heidary, M. Horch, I. Gudim, T. Simler, I. Zebger, O. Lenz, K. A. Vincent, *J. Phys. Chem. B* **2015**, *119*, 13807–13815.

[40] F. Roncaroli, E. Bill, B. Friedrich, O. Lenz, W. Lubitz, M.-E. Pandelia, *Chem. Sci.* **2015**, *6*, 4495–4507.

[41] J. Fritsch, E. Siebert, J. Priebe, I. Zebger, F. Lendzian, C. Teutloff, B. Friedrich, O. Lenz, *J. Biol. Chem.* **2014**, *289*, 7982–7993.
